# Colorectal cancer stem cells crosstalk in tumor immune microenvironment and targeted therapeutic strategies

**DOI:** 10.3389/fimmu.2025.1714954

**Published:** 2025-11-19

**Authors:** Yuxuan Duan, Anshu Li, Daojia Miao, Diaoyi Tan, Keshan Wang, Jian Shi

**Affiliations:** 1 Institute of Urology, Union Hospital, Tongji Medical College, Huazhong University of Science and Technology, Wuhan, Hubei, China; 2 Department of Gastrointestinal Surgery, Union Hospital, Tongji Medical College, Huazhong University of Science and Technology, Wuhan, Hubei, China; 3 Department of Urology, Union Hospital, Tongji Medical College, Huazhong University of Science and Technology, Wuhan, Hubei, China

**Keywords:** colorectal cancer stem cells, colorectal cancer, tumor immune microenvironment, immunosuppression, immunotherapy

## Abstract

Colorectal cancer (CRC) remains a formidable clinical challenge due to therapy resistance, metastasis, and relapse. Central to these processes are colorectal cancer stem cells (CCSCs), a dynamic subpopulation endowed with self-renewal capacity, plasticity, and heterogeneity. This review synthesizes recent advancements in understanding how CCSCs orchestrate tumor progression through intricate bidirectional crosstalk with the tumor immune microenvironment (TIME). We begin by elucidating the cellular origins of CCSCs, their profound intratumoral heterogeneity, and their remarkable phenotypic plasticity—driven by genetic, epigenetic, and metabolic reprogramming—which collectively serve as the root cause of therapeutic failure. A significant portion of our discussion is dedicated to deconstructing the immunosuppressive niche co-opted by CCSCs. We detail mechanisms of immune evasion and tolerance, highlighting how CCSCs modulate innate and adaptive immune cells—including NK cells, Tregs, dendritic cells, macrophages, neutrophils, and myeloid-derived suppressor cells—to foster an environment that supports stemness and suppresses cytotoxic attack. This reciprocal interaction forms a vicious cycle that perpetuates tumor survival and progression. Finally, we critically evaluate emerging therapeutic strategies that concurrently target CCSC-specific vulnerabilities and counteract immunosuppression. We explore the limitations of conventional chemotherapy and the promise of targeted therapies (e.g., Wnt inhibitors), immunotherapies (e.g., CAR-T, bispecific antibodies), and combination regimens designed to remodel the TIME and eradicate the CCSC reservoir. By integrating insights from single-cell omics and spatial biology, this review provides a comprehensive framework for overcoming therapy resistance and proposes novel precision medicine approaches for CRC.

## 1 Introduction

Colorectal cancer (CRC) ranks as the third most common malignant tumor and the second leading cause of cancer death worldwide (1). In recent years, its incidence has shown a trend toward younger age groups, with the rate among individuals under 50 increasing by 2% annually, becoming a significant public health concern (2). Despite continuous advancements in comprehensive treatment approaches such as surgery, chemoradiation, and immunotherapy, the prognosis for CRC patients remains unfavorable. Approximately 30%–40% of patients experience metastasis within several years after radical resection of the primary tumor (3).

Tumor metastasis, recurrence, and drug resistance are the primary causes of treatment failure (4, 5). Among these, “Intratumoral Heterogeneity (ITH)” is considered one of the key factors driving these malignant processes (6, 7). For example, within the same tumor mass, KRAS-mutated subpopulations exhibit intrinsic resistance to EGFR inhibitors (8), while other clonal subpopulations with cancer stem cell characteristics can enter a reversible G0 quiescent state upon re-exposure to elevated 5-FU concentrations (9). These functionally diverse subpopulations not only synergistically drive tumor evolution but also provide “adaptive reserves” for recurrence and distant metastasis. Consequently, higher levels of ITH in CRC correlate with increased biological malignancy, accelerated disease progression, heightened risk of recurrence and metastasis, and poorer patient prognosis (10).

Current research indicates that Cancer Stem Cells (CSCs) are considered the core driver subpopulation of tumor heterogeneity (10–12). Research on Colorectal Cancer Stem Cells (CCSCs) began in the early 21st century, when scientists successfully isolated and identified a subpopulation of cells with stem cell characteristics from CRC tissues. These cells express specific surface markers such as CD133, CD44, and Lgr5, and exhibit potent tumorigenicity, drug resistance, and metastatic potential compared to conventional cancer cells (13, 14).

Although eliminating CCSCs is considered an ideal strategy for radical tumor eradication and preventing recurrence and metastasis, numerous challenges persist in practical application. On one hand, CCSCs typically exist in a low-proliferative or dormant state, making them difficult to eradicate with traditional chemotherapeutic agents that rely on rapid cell division. On the other hand, CCSCs exhibit significant dynamism and plasticity; non-stem cell subpopulations can revert to CSC-like phenotypes under specific microenvironmental stimuli, leading to continuous renewal and maintenance of the stem cell phenotype. Consequently, therapeutic strategies targeting CSCs often fail to achieve complete efficacy, leading to the designation of CSCs as the “root cause of tumor persistence.”

Therefore, building upon Paget’s “seed and soil” concept, therapeutic strategies that target the “soil”, which refers to the tumor immune microenvironment (TME), have been developed. These approaches, such as combination therapies involving CAR-T and immune checkpoint inhibitors, are designed to enhance T cell-mediated cytotoxicity against tumor cells. However, due to the significant tumor heterogeneity in CRC, the “soil” states vary considerably, with differing degrees of immune cell infiltration. This ultimately results in a “polarized” response to immunotherapy: MSI-H/pMMR-type patients (TMB-high, inflammatory microenvironment) achieve favorable outcomes (15); MSS/pMMR type (TMB-low, immune-rejecting microenvironment) exhibits poor prognosis due to immune tolerance and immune escape. This is attributed to insufficient antigen presentation caused by low TMB, reduced CD8+ T cell infiltration, and increased immune-suppressive cells (16).

Furthermore, studies have revealed that crosstalk between CCSCs and immune cells significantly enhances immunosuppression [17]; conversely, activated immunosuppressive cells can amplify the stemness characteristics of CCSCs [18], forming a self-reinforcing “vicious cycle.” Therefore, unraveling the intricate interplay between CCSCs and the immune microenvironment to sever this vicious cycle and reverse immune suppression is recognized as a critical strategy for overcoming tumor recurrence and metastasis while enhancing therapeutic efficacy.

In recent years, CSCs have garnered significant attention as key drivers of tumorigenesis, metastasis, recurrence, and treatment resistance. However, the majority of current studies predominantly focus on the general properties of CSCs, whereas the mechanisms through which specific cancer stem cell subpopulations, especially CCSCs, contribute to immune regulation remain inadequately elucidated and have not been systematically investigated. This paper aims to comprehensively summarize the latest research advances on CCSCs, exploring their origin, heterogeneity, plasticity, and biomarkers. It will also investigate how interactions between CCSCs and immune cells promote the maintenance of cancer stemness, thereby driving disease progression and reinforcing the immunosuppressive microenvironment. Finally, we will outline future research directions and potential intervention strategies, seeking to provide new insights for achieving precision treatment in CRC.

## 2 Stem cell origin and characteristics of colorectal cancer

### 2.1 Origin

CCSCs are considered core drivers of tumorigenesis, recurrence, and drug resistance. In recent years, with the advancement of cutting-edge technologies such as single-cell sequencing, organoid culture, and lineage tracing, the origin mechanisms of CCSCs have gradually been elucidated. The initially identified CRC cells originated from stem cells in the basal layer of intestinal crypts (expressing markers such as LGR5, BMI, or CD133). These cells transformed into tumor-initiating cells under the sustained activation of the Wnt/β-catenin signaling pathway mediated by APC gene mutations (17).

Subsequent studies demonstrated that CRC can originate from differentiated cells, primarily driven by NF-κB structural activation. Additionally, research indicates that under the combined effects of APC gene loss and inflammatory stimulation (such as chemically induced colitis), CRC may also arise from a quiescent DCLK1-positive crypt cell subpopulation in a differentiated state (18–20).In MSS/pMMR-type CRC, Chen, B et al. (2021) employed single-cell transcriptomics (scRNA-seq) analysis to demonstrate that this subtype primarily arises from malignant transformation of stem cells at the crypt base (21). Subsequently, Mzoughi S et al. (2025) discovered that differentiated cancer cells can reacquire a human progenitor-like state through cancer-embryo reprogramming (22); MSI-H/dMMR CRC may arise from differentiated cells in the crypt epithelium undergoing dedifferentiation to acquire stem-like molecular characteristics before becoming malignant (23). These studies indicate that crypt basal stem cells are not the sole origin of CRC; differentiated cells can also reacquire stemness to promote cancer initiation and progression. Consequently, attention has shifted to the critical role of the microenvironment in cell fate determination, prompting exploration of crosstalk between CCSCs and their microenvironment.

Although conventional perspectives have long regarded CCSCs as a static cell population with well-defined phenotypic and molecular profiles, research conducted over the past two decades has increasingly demonstrated that CCSCs actually represent a dynamically evolving cellular entity, which can be more accurately described as a transient stemness state, whose plasticity is modulated by factors such as the tumor immune microenvironment (24).

### 2.2 Heterogeneity

ITH manifests as significant differences in genetic, epigenetic, and functional characteristics among cell subpopulations within the same tumor. This heterogeneity poses a barrier to effective treatment: higher levels of ITH increase susceptibility to drug resistance, accelerate tumor progression, and ultimately lead to poorer patient outcomes (10).

Tumor heterogeneity can primarily be categorized into three types: genetic heterogeneity, epigenetic heterogeneity, and functional heterogeneity.

Genetic heterogeneity arises from differential mutations in driver genes. For instance, CSCs may accumulate mutations during division due to DNA repair defects or epigenetic dysregulation, giving rise to subclones with distinct genetic backgrounds. For example, APC-mutated CSCs may further acquire KRAS or TP53 mutations, activating different signaling pathways and leading to the formation of ITH (25). Epigenetic heterogeneity modulates gene expression through DNA methylation or histone modifications, further exacerbating cellular phenotype differentiation. This generates mesenchymal phenotype subpopulations with enhanced migration and invasiveness (activated epithelial–mesenchymal transition(EMT), high expression of Vimentin and ZEB1), making them more likely to detach from the primary tumor site, metastasize to the liver or lungs, and form metastatic lesions (26). Functional heterogeneity manifests as the coexistence of stem-like subpopulations and differentiated cells. KRAS-mutant subpopulations exhibit intrinsic resistance to EGFR inhibitors (e.g., cetuximab) (8) while CD133+ cells evade apoptosis and retain stem-like properties (27).

Therefore, understanding the cellular composition of ITH and its underlying regulatory mechanisms is crucial. With the rapid advancement of bioinformatics technologies such as single-cell sequencing, researchers have employed scRNA-seq and functional analysis to progressively elucidate how ITH is established during early cancer development and how intratumoral cell subtypes dynamically evolve during progression. Based on 10× single-cell transcriptomics, seven subpopulations—including the CCSCs themselves—were identified within xenografts derived from CCSCs: The T1 cluster, associated with cell migration (highly expressing KRT19, MMP7, and TSPAN8); the T2 cluster, linked to endoplasmic reticulum stress (highly expressing HSPA1B, HSPA1A, and DUSP1); the T3 cluster, related to proliferative potential (highly expressing Ki67, CCNB1/2, and TOP2A); T4 cluster, associated with ciliary assembly and response to stimulation (high expression of AGR2, AGR3, and SNTN); T5 cluster, associated with response to hormones (high expression of ODC1, SELK, and CREM); T6 cluster, associated with apoptosis regulation (high expression of ANXA1, HEPACAM2, and ANXA4). These subpopulations exhibit distinct functional characteristics and dynamically emerge during xenograft tumor progression. Notably, T1, T3, and T4 subpopulations appeared as early as day 2, while the remaining subpopulations emerged by day 4. The proportion of each subpopulation gradually increased from day 2 to day 10. By day 12, the T3 and T5 subpopulations continued to increase, while the T2, T4, and T6 subpopulations decreased. These data indicate that different subpopulations generated by CCSCs undergo dynamic changes during xenograft progression: proliferative and invasive subpopulations emerge early in xenograft tumors. Tracking progeny cells generated via asymmetric division of CCSCs using Smart-seq2 revealed five subpopulations (C0–C4). Among these, C1/C2 constituted highly proliferative subpopulations, C3 represented a chemotherapy-resistant subpopulation (highly expressing CFAP54 and SEMA3E), and C4 constituted an invasive subpopulation (marked as PLAUR+). C0–C4 corresponded to certain T-series subpopulations (e.g., C0 to T4, C4 to T1) and emerged early in CRC development. T2, T5, and T6 were not detected in asymmetric division progeny, suggesting their generation likely originates from other mechanisms, such as genetic and epigenetic alterations, and they are crucial for late-stage ITH formation (10).

**Figure 1:**
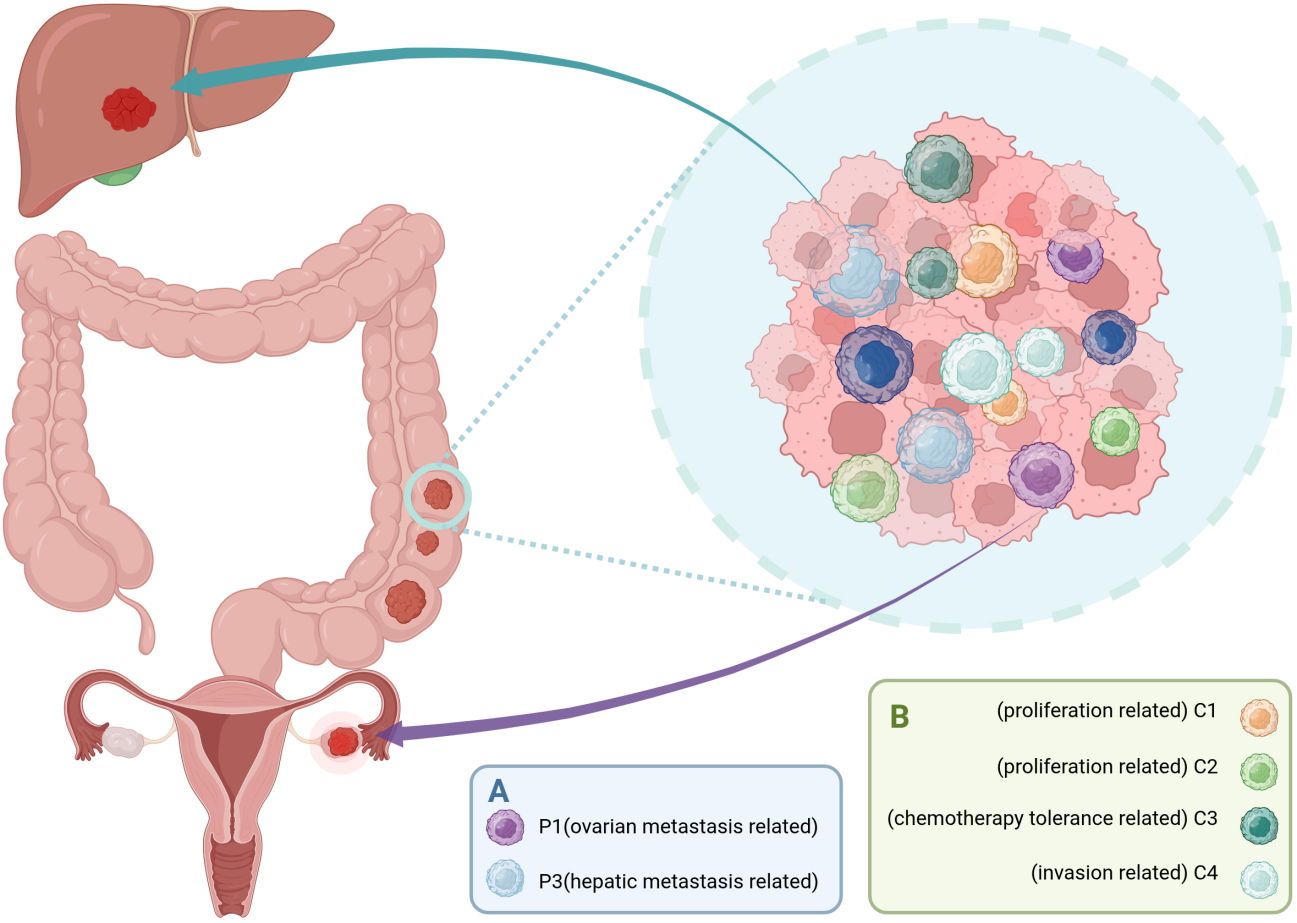
Distinct subpopulations of CCSCs drive organ-specific metastasis and functional intratumoral heterogeneity. **(A)** CCSCs pre-programmed for organ-specific metastasis. The P1 subpopulation is associated with ovarian metastasis, while the P3 subpopulation is linked to liver metastasis. **(B)** CCSCs give rise to progeny with diverse functional roles through processes such as asymmetric division. These include the highly proliferative C1 and C2 subpopulations, the chemotherapy-tolerant C3 subpopulations, and the invasion-prone C4 subpopulation. Together, these specialized CCSC subpopulations contribute to the multifaceted malignancy of colorectal cancer, including metastatic spread, therapy resistance, and tumor progression. (Created with BioRender.com).

Li et al. demonstrated through scRNA-seq and spatial transcriptomics analysis distinct metastatic-prone cancer stem cell subpopulations in CRC. Among these, P1 cells highly express delta-like ligand 4 (DLL4) and MAF bZIP transcription factor A (MAFA), enriched in both primary CRC and ovarian metastatic colorectal cancer (oCRC). They may drive ovarian metastasis by activating the NOTCH signaling pathway. In contrast, P3 cells display a gene expression profile resembling that of cholangiocytes, characterized by elevated levels of TOMM6, CXCL14, ATP6V0C, PSMA6, CALML4, DBNDD2, RNASE4, and DEFB1. This subpopulation is predominantly found in primary CRC and liver metastatic colorectal cancer (lCRC), indicating a specific tropism for liver metastasis (28) (**Figure 1**).

Research by Takeru Oka et al. using a syngeneic transplantation model of CRC organoids revealed that, based on scRNA-seq analysis, Lgr5+ cancer stem cells are divided into actively proliferating and quiescent populations, with the latter found to specifically express p57. In CRC, quiescent Lgr5+ CCSCs contribute minimally to tumor growth under stable conditions. However, they become activated by chemotherapy and significantly drive tumor regrowth. Knocking out p57+Lgr5+ cancer stem cells substantially reduces CRC recurrence after treatment (29, 30). In summary, these novel findings highlight the inherent heterogeneity of Lgr5+ cancer stem cells and underscore the necessity for novel therapeutic strategies targeting quiescent CCSCs to eradicate CRC (31).

In summary, CCSCs represent the root cause of intratumoral heterogeneity in CRC. Dynamic evolutionary studies reveal that CCSCs generate intrinsic heterogeneity early in tumorigenesis, further driving the establishment of ITH in CRC, ultimately leading to significantly reduced treatment efficacy. However, the formation mechanisms of distinct CCSCs subpopulations remain unclear: some may arise from asymmetric division (e.g., T1/T3 subpopulations), while others develop metastatic propensity through preprogramming and microenvironmental selection (e.g., P1/P3 organ-specific subpopulations). Future research urgently requires in-depth elucidation of these mechanisms to provide novel strategies for targeting the root causes of ITH (32–34).

### 2.3 Plasticity

Cell plasticity refers to the ability of cells to reprogram and alter their fate and characteristics (35). Recent studies indicate that CCSCs exhibit remarkable plasticity—they are not a stable, fixed population but exist in a state of dynamic cellular transition (31, 36, 37). This characteristic enables CCSCs to maintain stemness and adapt to microenvironmental changes. Through dynamic switching of phenotype and function, they drive therapeutic resistance, migration, invasion, and metastatic recurrence, serving as a key factor in the malignant progression of colorectal cancer.

The potent plasticity of CSCs enables them to switch between different cellular states. The plasticity of CCSCs primarily manifests in two directions: forward plasticity and reverse plasticity. In “forward” plasticity, CCSCs can differentiate from a stem cell state into various phenotypes such as mesenchymal, dormant, or drug-resistant states, fostering high intratumoral heterogeneity and conferring formidable invasiveness and adaptability to the tumor (12, 38). “Reverse” plasticity, or the process by which differentiated tumor cells regain stemness through “dedifferentiation,” ensures the persistent existence of stem cell populations (36). This reverse regenerative capacity allows stem cell populations to recover even after partial elimination, serving as a core mechanism for tumor persistence and recurrence and posing a significant clinical challenge.

It is now clear that the stemness of CSCs is coordinated by both genetic mutations and epigenetic mechanisms. In microsatellite-stable colorectal cancer driven by APC mutations, the APC mutation causes sustained nuclear translocation of β-catenin, activating stemness genes. Concurrently, cancer cells undergo Oncogenic Fetal (OnF) reprogramming driven by YAP and AP-1, achieving dedifferentiation through epigenetic regulation and reacquiring a state resembling human embryonic progenitor cells. This state is closely associated with tumor invasiveness and poor prognosis (22). Another team collected normal colon, primary tumor, and metastatic tissue samples from 31 CRC patients to establish organoid models. Through single-cell RNA sequencing and immunofluorescence staining analysis, they demonstrated that metastatic cancer cells also undergo OnF (39).

Compared to genetic mutations, epigenetic regulation serves as a more critical driving mechanism enabling CCSCs to achieve plasticity and adapt to environmental changes. Its core advantage lies in its ability to undergo targeted reprogramming in response to microenvironmental signals, thereby efficiently mediating dynamic transitions in cellular states.

TME signaling serves as a core dynamic switch regulating CCSC plasticity. Diverse signaling molecules within the TME induce the expression of cancer cell-associated signaling pathway transcription factors, thereby enabling CCSC plasticity. For instance, TAMs activate stemness pathways via IL-1β, IL-6, and TNF-α, with downstream activated transcription factors regulating stemness gene expression through epigenetic mechanisms, restoring quiescent Dclk1+ cells to an actively dividing stem cell state (40). Cancer-Associated Fibroblasts (CAFs) secrete miR-21 into tumor cells, activating the Wnt pathway to promote stemness, invasiveness, and drug resistance (41).

Metabolic reprogramming serves as the core driver of plasticity in CCSCs, dynamically adjusting energy and material metabolism to adapt to microenvironmental changes, thereby supporting their state transitions. Colorectal cancer cells adapt to fluctuating energy demands through glycolytic reprogramming to support biosynthesis and dedifferentiation (42). When intra-tumoral glutamine depletion causes a sharp drop in α-KG, TET/JmjC enzymes are inactivated, and stemness gene promoters undergo rapid hypermethylation, forcing colorectal cancer cells to exit the stem state toward differentiation or transition into dormant drug resistance (43). Concurrently, oncogenic APC mutations drive excessive accumulation of free cholesterol in the plasma membrane, significantly increasing membrane rigidity. This physical remodeling promotes the aggregation of Wnt receptors into signaling hubs within lipid rafts, sustaining and amplifying Wnt signaling. This further drives the maintenance of stemness and malignant progression in tumor cells (44).

It can be said that the plasticity of CCSCs endows tumor evolution with infinite possibilities and is one of the root causes of CCSCs’ heterogeneity. Research on plasticity has shattered the traditional notion that “targeting a fixed stem cell subpopulation achieves a cure,” yet it represents the core hope for breaking through current therapeutic bottlenecks. Targeting plasticity regulatory nodes holds promise for simultaneously dismantling the foundations of stem cell reservoir renewal and heterogeneity construction, offering a new paradigm for curative therapies.

## 3 Colorectal cancer stem cells markers

Identifying specific markers that distinguish CCSCs from most cancer cells holds significant scientific and clinical implications for colorectal cancer treatment. To date, researchers have identified a series of molecules closely associated with stemness maintenance across multiple levels, ranging from membrane receptors and transcription factors to long non-coding RNAs (45). **Table 1** summarizes currently widely recognized core markers and their primary functions in CCSCs. Among these, LGR5 acts as an amplifier of Wnt/β-catenin signaling, sustaining cellular self-renewal through continuous activation of the canonical Wnt pathway; CD133 regulates membrane microdomains while directly activating the PI3K/Akt axis and indirectly amplifying Wnt signaling to consolidate stemness; the “adhesion-migration” duo CD44 and CD24 mediates cell-matrix interactions and enhances tumor invasiveness by regulating immune evasion; Beyond sustaining stemness and self-renewal, EpCAM drives EMT to promote metastasis. At the nuclear transcription level, KLF5 serves as the master stemness regulator, BMI1 acts as a differentiation suppressor, and pluripotency TFs like SOX2, Nanog, and c-Myc collectively form the core regulatory network of CCSCs.

**Table 1:** Markers of CCSCs.

Name of marker	Functional role	Function	references
LGR5	GPCR	Amplifies Wnt/β-catenin signaling to maintain stemness.	(46, 47)
CD133	Membrane microdomain regulatory protein	Directly activates PI3K/Akt and indirectly amplifies Wnt/β-catenin signaling.	(48, 49)
CD24	Membrane surface signaling protein	Indirectly promotes cell adhesion, migration, and immune evasion	(48, 50)
CD44	Cell surface adhesion molecule	Directly mediates cell adhesion, migration, and stemness maintenance	(48, 49, 51)
EpCAM	Cell surface adhesion molecule + Signaling protein	Maintains stemness and self-renewal; promotes EMT	(52–54)
KLF5	Transcription factor	Maintains stemness and self-renewal	(55)
SOX2			(56, 57)
Nanog			(56, 58)
c-Myc			(56, 59)
BMI1	Transcriptional repressor	Inhibits differentiation; maintains stem cell self-renewal	(60, 61)

LGR5 Leucine-rich repeat-containing G-protein coupled receptor 5, CD133 Prominin 1, CD24 CD24 molecule, CD44 CD44 molecule, EpCAM Epithelial cell adhesion molecule, KLF5 Krüppel-like factor 5, SOX2 SRY-box transcription factor 2, Nanog Nanog homeobox, c-Myc MYC proto-oncogene, BMI1 BMI1 proto-oncogene

A retrospective analysis of transcriptomic and clinical data from the public database (TCGA) demonstrated that CD133, CD24, and CD44 are all associated with stemness signaling pathways. Furthermore, the high CD44/low CD24 combination was significantly correlated with poorer disease-specific survival and higher recurrence risk [53]. Leng et al. identified Lgr5+CD44+EpCAM+ cells as exhibiting the strongest stem cell characteristics both *in vitro* and *in vivo* by flow-sorting distinct subpopulations from DLD-1 cells (a human colorectal adenocarcinoma cell line) (52).

Beyond these established markers, several other key molecules have emerged as critical regulators of CCSC properties.EPHB2 suppresses invasion by limiting excessive stem cell proliferation; OCT4-high cells possess self-renewal capability, overexpress CD44/CD133, and correlate with poor clinical prognosis; knockdown of TRIB3 significantly reduces tumor burden in mouse colons; REC8 enhances stemness and accelerates metastasis via the BTK/Akt/β-catenin axis; ZNF217 directly activates Notch1 signaling, amplifying Notch pathway output to maintain the CCSC population. Additionally, PrPc induces EMT via the MAPK1 pathway, while POLR1A promotes rapid CCSC proliferation by driving ribosomal biogenesis. The emergence of single-cell sequencing and spatial transcriptomics technologies holds great promise for uncovering novel molecules associated with CCSCs, providing a powerful platform to validate and explore candidates like those listed in **Table 2** (**Table 2**).

**Table 2:** Potential markers of CCSCs.

Name of marker	Function	references
EPHB2	Regulates stem cell proliferation and differentiation; associated with invasiveness in CRC.	(62)
OCT4	Upregulated in CRC; associated with self-renewal, differentiation, metastasis, and drug resistance of CSCs	(63–65)
TRIB3	Knockdown of TRIB3 reduces colon tumorigenesis in mice, the migration of CRC cells, and the growth of xenograft tumors.	(66)
REC8	Promotes metastasis in CRC by enhancing stemness via the BTK/Akt/β-catenin pathway	(67)
ZNF217	Directly targets and activates Notch1, enhancing Notch signaling to promote self-renewal and stemness marker expression in CRC stem cells	(68)
PrPc	Promotes EMT via the MAPK1 pathway	(69)
POLR1A	Drives ribosome biogenesis and promotes cell proliferation	(70)

EPHB2 Ephrin type-B receptor 2, OCT4 Octamer-binding transcription factor 4, TRIB3 Tribbles homolog 3, REC8 Meiotic recombination protein REC8 homolog, ZNF217 Zinc finger protein 217, PrPc Cellular prion protein, POLR1A RNA polymerase I subunit A

## 4 CCSC–immune system interactions

In CRC, the role of the immune system has evolved from a mere “defender” to an “actor” engaged in complex interactions with tumor stem cells, with CCSCs at the core of this transformation. A “bidirectional dynamic interaction” exists between CCSCs and the immune system: early tumors activate innate immunity but evade it by downregulating immune recognition molecules or secreting inhibitory cytokines (71); Subsequently, the tumor microenvironment recruits and polarizes suppressive immune cells, further suppressing effector T cell function. Additionally, certain immune cells release molecules that maintain CCSCs’ stemness (72, 73); Concurrently, both tumor cells and immune cells highly express immune checkpoint molecules, triggering inhibitory signaling that leads to T cell exhaustion. This ultimately establishes an immunosuppressive microenvironment that facilitates tumor escape.

Notably, the CRC immune microenvironment exhibits high heterogeneity, with significant differences in immune cell composition, CCSCs’ stemness characteristics, and metabolic states across distinct tumor regions. This heterogeneity contributes to the complexity of CCSCs’ role in shaping the immune microenvironment. In-depth analysis of this interaction network not only helps reveal novel immune escape mechanisms underlying CRC recurrence and metastasis but also provides new insights for precision therapies targeting the CCSC-immune axis.

Future research should focus on elucidating the differential signaling pathways of CCSCs across distinct immune microenvironment subpopulations. By integrating single-cell sequencing and spatial transcriptomics technologies, we aim to dissect the intricate mechanisms governing interactions between colorectal cancer stem cells and immune cells. This will accelerate the translation of immune-regulatory targeted therapies for colorectal cancer stem cells from fundamental research to clinical implementation.

### 4.1 Immune escape and immune tolerance

In fact, the reason malignant tumors can proliferate unchecked within the body lies not only in the cancer cells’ vigorous ability to divide, but also in their capacity to employ sophisticated strategies to evade surveillance and attack by the immune system —they either disguise themselves as normal cells, tricking the body’s immune system into recognizing them as “legitimate” or release signals that interfere with immune cell function. This renders the immune cells responsible for eliminating abnormal cells functionally paralyzed, preventing them from fulfilling their defensive duties.

Mechanisms of tumor immune evasion share intriguing similarities with maternal-fetal immune tolerance during pregnancy, as both involve active modulation of the immune system to avoid attacking “non-self” entities. A recent study published in *Cell* revealed that the immune checkpoint molecule B7-H4 is highly expressed in both the tumor microenvironment and placental extravillous trophoblasts. By interacting with maternal CD8^+^ T cells, B7-H4 induces functional exhaustion, thereby protecting the fetus from immune attack (74). Maternal-fetal immune tolerance demonstrates greater stability compared to tumor immune evasion, and comparative analysis of these processes may provide novel insights into how tumor cells evade immune surveillance.

The immune tolerance mechanism during pregnancy exhibits enhanced stability. Comparative analysis of the mechanisms underlying tumor immune escape and pregnancy-induced immune tolerance may offer new insights into how tumor cells evade immune surveillance. This research lays a crucial foundation for intervening in the tumor-suppressive immune microenvironment and disrupting its immune escape state.

Compared to conventional colorectal cancer cells, MHC Class I molecule expression is significantly downregulated in CRC cells. This downregulation is directly associated with defects in Antigen Processing Machinery (APM) molecules—APM-related molecules in CRC cells (such as HLA heavy chains, β2 -myosin, etc.) are generally downregulated or absent in CCSCs, leading to reduced antigen processing and presentation efficiency, which in turn affects the cell surface expression of MHC Class I molecules. By downregulating MHC Class I, CCSCs reduce the presentation of tumor antigens to T cells, thereby evading recognition and attack by CD8+ T cells. This represents one of their key strategies for immune escape.

Concurrently, CCSC cells highly express interleukin-4 (IL-4) on their surfaces. When T cells approach, IL-4 directly inhibits T cell proliferation and activity through direct contact, effectively “applying the brakes” to T cells (75).

For example, compared to LGR5+, LGR5− cells exhibit lower MHC-I expression, thereby evading immune surveillance. Multi-omics analysis of intestinal organoids demonstrated that following mutation acquisition, tumor-initiating LGR5+ stem cells begin expressing the transcription factor SOX17 (76), initiating a developmental regulatory program associated with fetal intestinal development. Driving cells to differentiate away from LGR5+ tumor cells and form a population of LGR5++− tumor cells with immune evasion properties (LGR5++− represents the transition from immune-sensitive LGR5+ to immune-evading LGR5− states within the tumor cell population). This enables tumor stem cells to achieve immune evasion through heterogeneous differentiation (77, 78).

**Figure 2:**
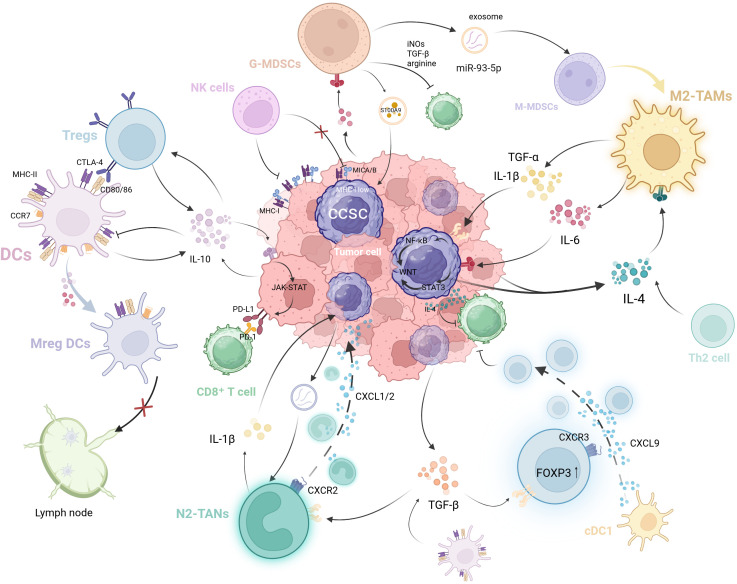
The self-reinforcing, bidirectional crosstalk between CCSCs and immune cells in the TIME. CCSCs evade immune surveillance through mechanisms such as MHC-I downregulation and immunosuppressive cytokine secretion (e.g., IL-4). They further remodel the microenvironment by recruiting and educating immunosuppressive cells via exosomes and chemokines. In return, these immune cells enhance CCSC stemness: TAMs secrete factors like IL-1β and IL-6, while MDSCs promote stemness through exosome S100A9. Critically, these immune cells form an interconnected network where Tregs suppress DCs function, MDSCs drive M2 macrophage polarization, and tolerogenic DCs promote Treg differentiation. This multicellular crosstalk establishes a vicious cycle that drives immune evasion and tumor progression. (Created with BioRender.com).

Beyond the intrinsic alterations occurring within CCSCs themselves, the direct interactions between CCSCs and immune cells make a significant contribution to the sustained growth and metastatic recurrence of CRC. Concurrently, the interactions among immune cells themselves exert important indirect effects, constituting a crucial component of the tumor-suppressive immune microenvironment (**Figure 2**).

### 4.2 NK cell

NK cells, a subset of cytotoxic lymphocytes, play a vital role in the innate immune system. These cells are highly proficient at detecting and eliminating tumor cells, making them key players in the body’s defense against malignancy.

It has been demonstrated that activated NK cells are capable of preferentially killing the CSC populations in breast cancer (CD24−/CD44+), pancreatic cancer (CD24+/CD44+), and glioblastoma (CD133+) (79–81). This selective cytotoxicity in NK cells against CSCs stems from two key factors: CSCs’ reduced MHC-I expression and increased NK-activating ligand levels. Notably, CSCs overexpress MICA/MICB, ligands for the NKG2D receptor on NK cells [The NKG2D pathway plays a central role in NK cell killing of tumor cells (81)].

As previously discussed, LGR5+ colorectal cancer stem cells upregulate the transcription factor SOX17, which suppresses expression of the stemness marker LGR5. This activates fetal intestinal development-related programs, leading to significant downregulation of interferon-γ receptor 1 (IFNγR1) and MHC-I at the transcriptional level. Interestingly, unlike the aforementioned cancer types, in colorectal cancer, despite exhibiting a low MHC-I expression phenotype, these SOX17+ tumor stem cells do not trigger enhanced NK cell infiltration and activation. J et al. analyzed that this occurs because mutated stem cells lack essential NK cell activation ligands while maintaining low-level rather than complete absence of MHC-I expression. This creates a “Goldilocks effect” similar to hair follicle stem cells—where MHC-I expression is just sufficient to evade NK cell recognition while avoiding CD8+ T cell immune surveillance, forming a dual escape strategy from adaptive and innate immunity (78).

### 4.3 TREG cells

Treg cells are a type of CD4+ T cell with immunosuppressive functions, primarily responsible for maintaining immune tolerance and tissue homeostasis while preventing autoimmune reactions. However, during cancer development, these normal physiological mechanisms of Treg cells can be exploited by tumor cells, thereby aiding tumor escape from immune system attacks and promoting tumor growth and progression (82). Treg cells produce large amounts of TGF-β and IL-10 to suppress both adaptive and innate immunity (83). A research team demonstrated in murine models that IL-10 acts on Tregs in an autocrine manner, creating a positive feedback loop to amplify IL-10 signaling. This autocrine activation subsequently induces PD-L1 upregulation in monocytes within the tumor microenvironment. Ultimately, the PD-L1/PD-1 axis dampens CD8^+^ T cell-mediated cytotoxicity against metastatic lesions, particularly in the liver, thereby facilitating hepatic metastasis formation (84). Additionally, high levels of cytotoxic T lymphocyte-associated protein 4 (CTLA-4) on Treg cell surfaces downregulate co-stimulatory molecules on antigen-presenting cells and induce tolerogenic dendritic cells, thereby suppressing immune responses (85).

Most current research focuses on the indirect relationship between CCSCs and Tregs within the tumor microenvironment. For instance, Tregs influence the function and maturation of dendritic cells (DCs) through interaction, thereby indirectly regulating the immune microenvironment of CCSCs. Studies on the direct interaction between these two cell types remain relatively scarce. Kono et al. demonstrated that hypoxia induces IL-17 expression in FOXP3^+^ Tregs. This IL-17 then drives Akt and MAPK signaling in CRC, subsequently facilitating the expansion of CSCs, which is characterized by increased expression of CD133, CD44, and EpCAM (86). This finding suggests that further in-depth research is needed to elucidate the mechanisms underlying the direct interaction between CCSC and Treg, offering new perspectives and strategies for colorectal cancer treatment.

### 4.4 Dendritic cells

Dendritic cells (DCs) are considered the most potent antigen-presenting cells, capable of recognizing, engulfing, processing antigenic peptides, and presenting them to helper T cells and cytotoxic T cells to stimulate their activation and proliferation, playing a crucial role in antitumor immune responses (87). Tumor-associated dendritic cells (TADCs) constitute 2.1% of immune cells in the CRC microenvironment, migrating between tumor and lymphoid tissues while recognizing and processing Tumor-Associated Antigens (TAAs) (88). In CRC, CD133+ CSCs impair the function of DCs by reducing the quantity of activated DCs (89).

Current research in other cancer types has revealed that the identity of some DCs shifts from promoting “immune killing” to facilitating “immune suppression.” DCs can secrete TGF-β to activate the Smad signaling pathway, thereby promoting the expression of the transcription factor FoxP3 and driving the functional differentiation of Tregs (90). Simultaneously, high IL-10 expression suppresses DCs’ own antigen presentation capacity (e.g., by downregulating MHC class II molecules and co-stimulatory molecules CD80/CD86). This synergizes preferentially with TGF-β to induce FoxP3^+^ Treg generation while inhibiting effector T cell (Th1/Th17) differentiation, rather than activating effector T cells (91, 92). Beyond autocrine TGF-β and IL-10, tumor cells in the microenvironment also downregulate DC surface MHC-II and CCR7 expression by secreting IL-10, IL-6, and others, thereby disrupting antigen presentation (93). Tregs also significantly suppress antigen presentation by cDC-2 and block their ability to induce CD4+ T cell differentiation (94). Consistent with this, a recent study demonstrated that cDC-1 recruits Tregs via the CXCL9-CXCR3 axis and promotes their activated phenotype to locally suppress CD8+ T cells in tumors (95).

Additionally, a research team utilized the ApcMin/+ mouse model to reveal that Mreg DCs, characterized by high expression of MHC class II molecules and co-stimulatory molecules, promote Treg activation through MHC II-TCR signaling. Activated Tregs suppress Mreg DCs via CTLA-4 and migrate to draining lymph nodes, thereby limiting tumor antigen presentation and impeding the initiation of anti-tumor adaptive immune responses. This mechanism was validated in human colorectal cancer samples, where Treg-Mreg DCs interactions were found to correlate with poorer patient prognosis (96).

CCSCs impair conventional DCs’ function not only by reducing DC abundance but also by skewing them toward a tolerogenic phenotype that favors Treg cells induction over effector T-cell priming (89, 90, 95, 97). Tregs, in turn, suppress DC antigen presentation through various mechanisms, forming a “positive feedback” pathway that generates immunosuppression. This protects cancer stem cells from immune-mediated killing. Overall, CCSCs and immune cells jointly promote the formation of an immunosuppressive microenvironment.

### 4.5 Macrophages

Macrophages are key components of the innate immune system, differentiating from monocytes in the bloodstream and helping the host combat inflammation and tumors (98, 99).In response to external environmental stimuli, macrophages recruit monocytes from the bloodstream to migrate to tumor sites and prompt their polarization into tumor-associated macrophages (TAMs) (100). Macrophages are typically categorized into two major subtypes: M1 macrophages with pro-inflammatory properties and M2 macrophages with anti-inflammatory functions (101).TAMs are the most abundant immune cells in the TME (13).TAMs typically exhibit an M2 phenotype, while under induction by IL-4/IL-10/IL-13 or glucocorticoids, they secrete anti-inflammatory cytokines like IL-10/IL-1β and promote angiogenesis/tissue remodeling/injury repair/tumorigenesis (102–104).

Growing evidence suggests a bidirectional interaction between TAMs and CCSCs (19, 105, 106, 107). On one hand, TAMs play a critical role in the generation, maintenance, and immunosuppression of CCSCs. A team has demonstrated that the presence of M2-type TAMs promotes the generation of cancer stem cell-like phenotypes in both HCT116 and DLD-1 colon cancer cells. Further research indicates that interleukin-6 (IL-6) derived from TAM or exogenous sources promotes the generation of CSCs (105). Macrophage-secreted IL-1β, IL-6, and TNF-α can reprogram quiescent Dclk1+ cells back to an actively dividing stem cell state (40). TAMs maintain CSC self-renewal via cross-talk between STAT3 and NF-κB signaling axes (106). Constitutive NF-kB activation was reported to enhance Wnt activation and stem cell marker expression in mouse crypt cells (19). Additionally, the depletion of suppressive macrophages can significantly enhance the killing efficacy of CSCs mediated by T cells. This finding strongly corroborates the critical role of TAMs in the immunosuppression of CSCs (107).

On the other hand, CCSCs shape TME, establishing a reciprocal interaction with TAMs. When TAMs are exposed to IL-4 secreted by CD133^+^ CCSCs (108), the expression of phosphoglycerate dehydrogenase (PHGDH) is upregulated, thereby promoting the polarization of TAMs toward an M2 phenotype. It is worth noting that in addition to CCSCs, Th2 cells are also a significant source of IL-4 (109). Upon binding to its receptor, IL-4 promotes the phosphorylation of STAT6, subsequently inducing M2-like macrophage polarization via the JAK/STAT6 signaling pathway (110). However, systematic research on the direct regulatory effects of CCSCs on TAMs remains limited. Wang et al. demonstrated that in CRC cells, CXCR4 upregulation mediates the transfer of specific miRNAs (miR-25-3p, miR-130b-3p, miR-425-5p) to macrophages via extracellular vesicles (EVs), thereby promoting M2 polarization through the PTEN/PI3K/Akt pathway (111). However, it remains to be confirmed whether CCSCs similarly utilize this EV-mediated mechanism to drive M2 polarization.

Neutrophils are phagocytes that constitute a vital component of the innate immune system and represent the earliest immune cells to appear during the acute phase of inflammation. These highly plastic cells undergo remodeling within the tumor microenvironment to become “Tumor-associated neutrophils (TANs).” TANs are polarized into cells exhibiting either N1 or N2 phenotypes. N1 cells suppress tumor cell proliferation and metastasis, while N2 cells assist tumor cells in evading immune surveillance, promote angiogenesis, and enhance tumor cell invasiveness (112, 113).

In CRC, stimulated by TNF-α, Granulocyte-macrophage colony-stimulating factor (GM-CSF), Platelet-activating factor (PAF), and CXCL8, neutrophils release CXCL8, CXCL1, and VEGF, inducing tumor angiogenesis (114). Purified Neutrophil extracellular traps (NETs) induce filopodia formation in colorectal cancer cells, thereby contributing to tumor metastasis. This correlates with increased expression of mesenchymal markers mRNA (vimentin, fibronectin) and pro-EMT transcription factors (ZEB1, Slug), alongside downregulation of epithelial cell adhesion molecule (EpCAM) and epithelial marker E-cadherin (CDH1) (115).

Recent studies have revealed that exosomes released by CCSCs carry 5′-triphosphate RNA, which acts as a transportable molecular pattern to activate the PRR–NF-κB signaling axis in neutrophils. This activation induces IL-1β expression, prolonging neutrophil survival and expanding the pro-tumor neutrophil pool. Furthermore, CCSCs secrete CXCL1/2 chemokines to recruit neutrophils into the TME, where IL-1β promotes tumorigenesis (116).

Activating the antitumor potential of neutrophils and utilizing them as antitumor effector cells represents a novel cancer treatment approach that has demonstrated promising efficacy in colorectal cancer (117). Suppressing pro-tumor neutrophils constitutes a new research direction in neutrophil-based cancer therapy. Within the TME, tumor-derived TGFβ can polarize TANs populations toward a pro-tumor phenotype. (118). Research has found that in co-cultures of neutrophils and SW480 cells (colon adenocarcinoma cells), neutralizing TGFβ with monoclonal antibodies inhibits cancer cell migration and enhances cytotoxicity of neutrophils targeting cancer cells (119–121). Moreover, NETs have been implicated as playing a crucial role in the advancement of cancer (122). Current research on anti-cancer drugs targeting NETs focuses on three main approaches: inhibition of NET formation, disruption of pre-formed NETs, and inhibition of interactions between cancer cells and NETs. In models of metastatic human breast cancer and colon cancer, Liang et al. used cationic materials derived from polyaspartic acid to competitively bind to NET-DNA with CCDC25, subsequently reducing the chemotactic potential of NET-DNA in cancer metastasis (123).

### 4.6 Myeloid-derived suppressor cells

Myeloid-derived suppressor cells (MDSCs) represent a population of pathologically activated neutrophils and monocytes, which exhibit powerful immunosuppressive activity. Under normal physiological conditions, the body contains low levels of MDSCs. When stimulated by inflammation, infection, tumors, or tissue damage, they proliferate extensively (124). In cancer, MDSCs promote tumor metastasis and assist tumor cells in immune evasion by suppressing immune cell function through multiple mechanisms. Additionally, during pregnancy, MDSCs participate in maintaining maternal-fetal immune tolerance (125). MDSCs are conventionally classified into two major subpopulations: polymorphonuclear (PMN-MDSCs, historically referred to as G-MDSCs) and monocytic (M-MDSCs) (126), which are phenotypically and morphologically analogous to neutrophils and monocytes, respectively (127).

Hypoxia can induce increased secretion from G-MDSC, which leads to enhanced stemness of CRC cells. G-MDSCs increase STAT3 phosphorylation, CD133 and CD44 expression, and sphere formation of CCSCs *in vitro* by secreting exosomes containing S100 Calcium-Binding Protein A9 (S100A9) under hypoxic stress (128). MDSCs secrete inhibitory molecules such as cathepsin, Inducible nitric oxide synthase (iNOS), and TGF-β, thereby suppressing effector T cell function (129, 130). During chronic intestinal inflammation, elevated IL-6 upregulates STAT3 activity in G-MDSCs via the IL-6R/JAK/STAT3 pathway, thereby increasing miR-93-5p transcription and subsequent enrichment in G-MDSC exosomes. G-MDSCs suppress STAT3 activity in M-MDSCs via exosomal miR-93-5p, thereby promoting M-MDSC differentiation into M2 macrophages. These mechanisms collectively create an immune-evasive environment for CCSCs, facilitating the transition from colitis to cancer (131).

A comprehensive analysis of existing literature indicates that immune cells within the TME exhibit plasticity rather than fixed immune characteristics. Under the influence of the microenvironment, they can differentiate into either antitumor or pro-tumor phenotypes. This phenomenon suggests that targeting a specific type of immune cell may be too broad an approach. Research on the interactions between immune cells and CCSCs remains relatively limited, and the specific interaction networks between them have not yet been fully elucidated. Therefore, focusing on studying these interactions is crucial for laying the foundation for future immunotherapies.

## 5 Therapeutic strategies targeting CCSCs and the tumor microenvironment

### 5.1 Chemotherapy

5-Fluorouracil (5-FU), an antimetabolite chemotherapy agent structurally similar to uracil, inhibits tumor cell proliferation by interfering with DNA and RNA synthesis, occupying a central role in CRC treatment. Despite the emergence of novel chemotherapeutic agents (e.g., irinotecan, oxaliplatin) and targeted therapies, 5-FU remains the cornerstone of CRC chemotherapy. Similarly, oxaliplatin, a third-generation platinum-based drug, serves as a first-line chemotherapy option for CRC. It primarily inhibits DNA replication and transcription by forming adducts upon binding to DNA.

Although traditional chemotherapeutic agents (such as 5-FU and oxaliplatin) demonstrate significant short-term efficacy in CRC treatment, their ability to eliminate CCSCs is limited. Drug resistance has become a critical bottleneck constraining therapeutic outcomes, ultimately potentially leading to tumor metastasis and recurrence. Research indicates that 5-FU chemotherapy activates the wild-type p53-mediated WNT3/β-catenin signaling pathway, enriching and activating LGR5+ CCSCs (132); simultaneously, 5-FU activates YAP signaling (rather than the conventional β-catenin pathway), driving the transformation of proliferative colonic stem cells (proCSCs) into dormant, reactivable stem colonic cells (revCSCs), enabling them to evade killing by entering dormancy. Although chemotherapy initially effectively eliminates highly proliferative proCSCs, revCSCs resist treatment and persist as residual disease. Upon cessation of treatment, these revCSCs can differentiate into proCSCs, rapidly proliferate, and cause tumor recurrence (37). Oxaliplatin has also been implicated in tumor resistance associated with EMT and CSCs (133).

As a key bottleneck in conventional therapies and the root cause of tumor recurrence and metastasis, research on CCSCs has made significant strides in recent years, driven by rapid advancements in technologies such as single-cell sequencing and organoid culture. Scientists have gained deeper insights into their molecular characteristics, regulatory mechanisms, and interactions with the tumor immune microenvironment (TIME), leading to the development of multiple novel targeted and immunotherapy strategies (134).

### 5.2 Targeted therapy

Constitutive activation of the Wnt/β-catenin pathway is a core driver of stemness maintenance and survival in CCSCs. Its molecular mechanism begins with Wnt ligands (such as Wnt3a) binding to cell membrane receptors (Frizzled/LRP5/6), triggering downstream signaling cascades. Upon pathway activation, the β-catenin degradation complex (comprising APC, Axin, and GSK3β) is inhibited. Mutations in the APC gene, present in over 80% of colorectal cancers, permanently disrupt this complex’s function, preventing cytoplasmic β-catenin from being phosphorylated and degraded. Accumulated β-catenin then translocates to the nucleus, binds to TCF/LEF transcription factors, and activates stemness genes such as LGR5, ASCL2, and c-MYC, driving CCSC self-renewal and drug resistance. Porcupine inhibitors block Wnt palmitoylation, causing unmodified Wnt proteins to accumulate in the endoplasmic reticulum and undergo degradation. This upstream inhibition of Wnt signaling significantly reduces β-catenin nuclear translocation, decreases stemness gene expression, and eliminates LGR5^+^ CCSC-enriched subpopulations (135).

Beyond this, Storm et al.’s study in Nature focused on colorectal tumors lacking APC mutations but harboring PTPRK-RSPO3 fusion genes: RSPO3 fusions sustain high expression of intestinal stem cell marker genes (such as LGR5 and ASCL2) by continuously activating the Wnt signaling pathway, thereby driving tumor stem cell function and tumor growth. Blocking RSPO3-mediated Wnt signaling using anti-RSPO3 antibodies disrupts the self-renewal capacity of tumor stem cells, leading to loss of tumor stem cell function. Moreover, its impact on normal intestinal tissue is limited due to functional redundancy between RSPO2 and RSPO3. This study provides experimental support for therapeutic strategies targeting CCSC-dependent pathways and offers a potential direction for personalized treatment of RSPO3 fusion-positive patients (136).

The significant overlap in molecular characteristics and signaling pathways between CCSCs and normal intestinal stem cells has resulted in limited efficacy of current targeted therapies specifically designed for CCSCs. Future research should prioritize identifying vulnerability targets unique to CCSCs that are absent in normal stem cells, thereby advancing the development of next-generation targeted therapies (71, 137).

### 5.3 Immunotherapy

Novel immunotherapy strategies targeting CSCs show great promise in enhancing anti-CSC effects. Representative approaches include: - Vaccines based on DCs and nanodisks (NDs) that present CSC antigens—such as CSC lysates, CSC marker proteins, or CSC-derived peptides—to induce anti-CSC immune responses; development of CSC-targeting bispecific antibodies (BiAbs) and antibody-drug conjugates (ADCs); and CSC-targeting chimeric antigen receptor (CAR)-T cell therapies and NK cell-based therapies, which have demonstrated progress in both solid tumors and hematologic malignancies (16, 138).

Based on the spatial distribution of CD8+ T cells within the TIME, the characteristics of immune cell infiltration, and the activation status of related signaling pathways, tumors are classified into three categories: immune-inflamed, immune-excluded, and immune-desert (139, 140). Immune-inflamed tumors are characterized by substantial CD8+ T cell infiltration into the tumor parenchyma with active function, typically responding well to immunotherapy. The latter two categories are considered “cold” tumors, manifesting as either ineffective CD8+ T cell infiltration into the tumor core (immune-excluded) or near-total absence of T cells within the tumor (immune-desert), rendering them largely unresponsive to immunotherapy (139, 141). In the context of CRC, this classification is particularly critical: CRC with high microsatellite instability/defective mismatch repair (MSI-H/dMMR) is definitively categorized as a “hot” tumor due to its unique, immune-cell-rich microenvironment. Conversely, microsatellite stable/probe mismatch repair normal (MSS/pMMR) CRC is primarily defined as a “cold” tumor (142).

Additionally, studies have found that metastatic colorectal cancer cell populations may contain a higher proportion of cells exhibiting cancer stem cell-like properties. Compared to primary colorectal cancer cells, these cells demonstrate greater sensitivity to NK cell-mediated lysis (143). Due to the widespread heterogeneity both between tumors and within tumors—meaning even primary and metastatic sites of the same cancer type exhibit significant differences in immune cell infiltration within their tumor microenvironments—this TIME heterogeneity directly leads to substantial variations in immune therapy responses across different tumor locations and even distinct regions of the same tumor. It is a key factor influencing the efficacy of immune therapy. Therefore, delving into TIME heterogeneity—particularly how it influences the recognition and killing efficacy of immune cells (such as NK cells and T cells) against tumor cells (especially CSC subpopulations), and how to overcome the immunosuppressive barriers of “cold” tumor microenvironments—is crucial for developing more precise and effective immunotherapy strategies.

Understanding the dynamic equilibrium of TIME is crucial. It can be viewed as a battlefield where two forces—the “offensive” and the “defensive”—engage in continuous struggle: the “offensive” aims to eliminate tumors through strategies such as enhancing effector T cell and NK cell function (e.g., CAR-T (144) and CAR-NK therapies (145)) or releasing T cell suppression (e.g., using immune checkpoint inhibitors like anti-PD-1/PD-L1) (146); while the “defense” promotes tumor survival. Tumor cells and cells like CCSCs suppress immune cell function by secreting immunosuppressive factors. For instance, TGFβ signaling plays a crucial role in enhancing immune resistance in colon cancer. This signaling promotes Treg differentiation and suppresses Th1 immune responses, thereby constructing a robust immune resistance barrier. Treatment with the TGFβ receptor inhibitor galunisertib significantly restores CD8^+^ T cell infiltration and activates Th1 immune responses, thereby inhibiting primary tumor growth and liver metastasis formation (71). Crucially, when galunisertib is combined with PD-L1 antibodies, synergistic effects are achieved through synchronous mechanisms: galunisertib weakens “defensive” immune suppression (releasing T cell blockade/inhibiting Treg activation), while the PD-L1 antibody enhances “offensive” cytotoxicity (reactivating T cell killing function). This strategy significantly extended survival in mouse models with established metastases, offering a breakthrough approach for immunotherapy resistance in MSS colorectal cancer patients. It fully validates the core value of “coordinated offensive-defensive intervention” in reshaping the immunosuppressive TIME (71). A 2023 clinical study reported that some patients intolerant to chemotherapy regimens achieved significant suppression of tumor recurrence after CAR-NK cell therapy, with no notable immune-related adverse events (145).

Current immunotherapy strategies primarily focus on enhancing the “offensive” approach, with their core limitation being the failure to effectively weaken the “defensive” forces—specifically, the inability to sufficiently eliminate or suppress immunosuppressive cells (such as Tregs and MDSCs) and soluble immunosuppressive molecules within the tumor microenvironment. Achieving effective remodeling of the immunosuppressive TIME, promoting the transformation of “cold tumors” into “hot tumors,” and making effective immunotherapy a viable option for a broader range of cancer types represent key current directions in immunotherapy (139).

### 5.4 Traditional Chinese medicine treatment

Traditional Chinese medicine, as the traditional medicine of the Chinese nation, demonstrates significant clinical application value in various diseases through its numerous active components. Research has revealed that multiple herbal constituents can target specific signaling pathways to inhibit CCSCs. For instance: caffeic acid and silibinin target the PI3K/Akt/mTOR signaling pathway; while honokiol and quercetin target the NOTCH signaling pathway. Furthermore, clinical practice demonstrates that traditional Chinese medicine can not only complement Western medical treatments to enhance efficacy and mitigate side effects but also be applied independently to achieve tumor growth control and prolong patient survival (147).

### 5.5 Integrative therapeutic model and rationale

**Figure 3:**
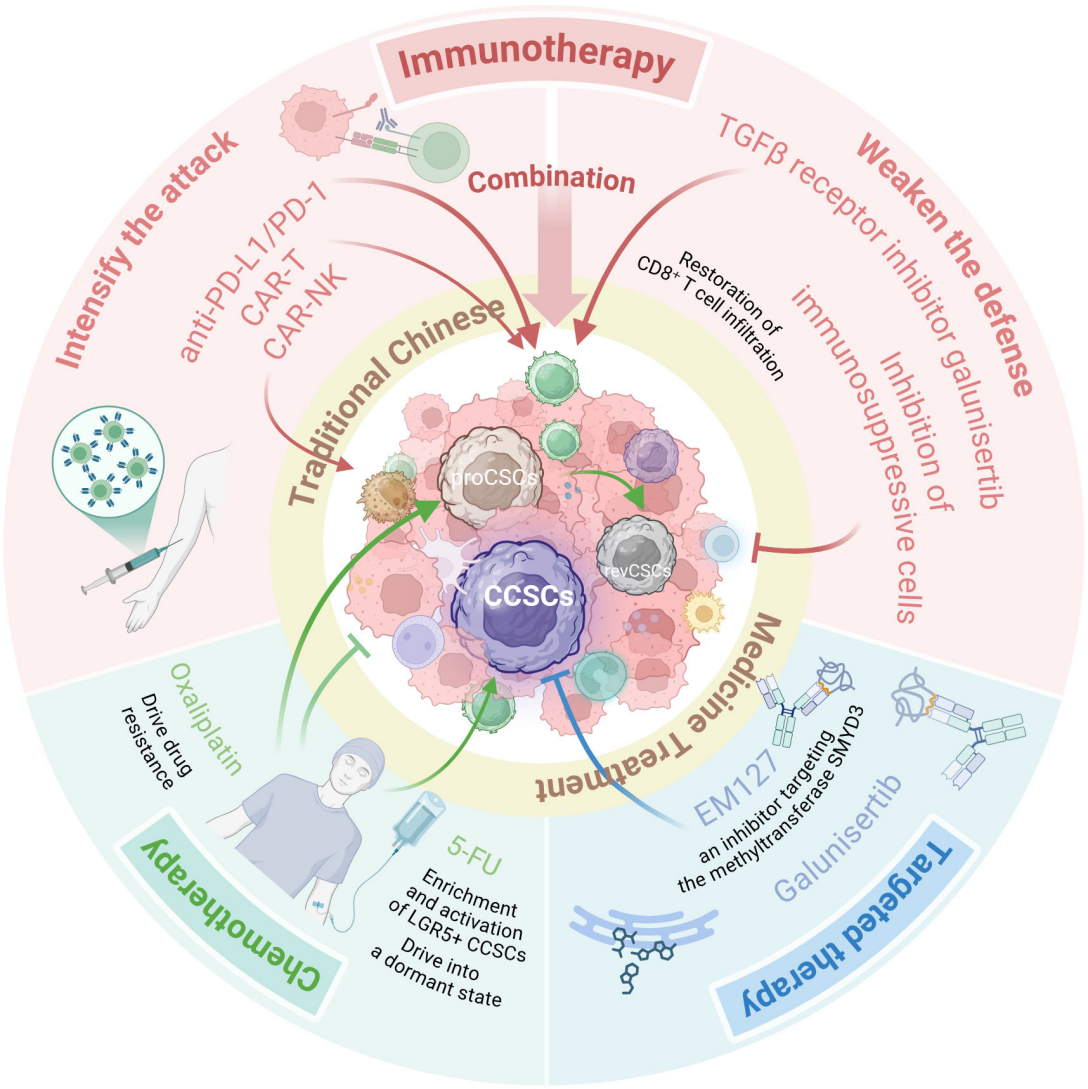
An integrative therapeutic model to eradicate CCSCs and overcome therapy resistance. This schematic illustrates a multi-modal strategy designed to disrupt the vicious cycle between CCSCs and the immunosuppressive TIME. The model synergizes several core approaches: Chemotherapy & Targeted Therapy (e.g., 5-FU combined with the SMYD3 inhibitor EM127) to reduce tumor burden and directly undermine CCSCs; Immunotherapy (e.g., combining TGF-β inhibitors with PD-1 inhibitors) to reverse immunosuppression and convert “cold” tumors into “hot” tumors; and Traditional Chinese Medicine, utilizing multi-targeting components (e.g., caffeic acid, silibinin, honokiol, quercetin) to suppress CCSC stemness pathways and mitigate treatment side effects. This combinatorial approach aims to achieve deep remission by simultaneously attacking intrinsic CCSC resistance mechanisms and extrinsic immune evasion barriers. (Created with BioRender.com).

Given the heterogeneity and plasticity of CCSCs and the vicious cycle they form with the TIME, monotherapies often yield limited efficacy in eradicating CCSCs. This has driven researchers to develop an integrated therapeutic paradigm centered on simultaneously targeting the intrinsic resistance mechanisms of CCSCs and the extrinsic immune escape barriers provided by TIME. A prospective therapeutic model aims to achieve deep remission through synergistic multi-mechanism action (**Figure 3**): First, chemotherapy or targeted drugs reduce overall tumor burden while directly undermining the stemness foundation of CCSCs. Concurrently, immune modulators are combined to reshape the TIME, reversing immunosuppression and transforming “cold tumors” into immune cell-rich “hot tumors.” Research confirms that EM127, an inhibitor targeting the methyltransferase SMYD3, combined with 5-FU chemotherapy, significantly reduces the proliferative capacity of colorectal cancer stem cells and induces their apoptosis. This combination achieved complete tumor regression in some tumor-bearing mice without observable significant side effects, demonstrating the superiority of the targeted therapy and chemotherapy combination regimen (148). As previously mentioned, the combination regimen of TGF-β inhibitors and PD-1 inhibitors in immunotherapy has demonstrated preliminary efficacy in MSS colorectal cancer (71). Of particular note, traditional Chinese medicine demonstrates unique value within this integrated model. Its diverse active components—such as caffeic acid and silibin targeting the PI3K/Akt/mTOR pathway, and magnolol and quercetin targeting the NOTCH pathway—can inhibit CCSC characteristics through multi-targeted action. Complementing Western medical treatments, these compounds enhance efficacy while mitigating toxic side effects, This approach holds promise for achieving deep remission in colorectal cancer and prolonging patients’ progression-free survival.

## 6 Discussion

With advances in colorectal cancer treatment, the primary challenge has shifted from “overcoming initial treatment failure” to “controlling recurrence risk.” CCSCs have been demonstrated to play a pivotal role in recurrence, metastasis, and treatment resistance (148). Recent studies progressively reveal that CCSCs are not static entities but rather plastic cellular states dynamically regulated by the TIME. Are CCSCs truly the “culprits”? Signaling molecules within the tumor immune microenvironment may be the “root cause.”

This review systematically elucidates: (1) CCSCs exhibit “bidirectional plasticity,” achieving reversible transitions between stemness and differentiation states through epigenetic reprogramming (e.g., OnF embryonic-like state, EMT conversion). CCSCs serve as the root cause of ITH, and their intrinsic subpopulation heterogeneity drives organ-specific metastasis and recurrence (10). (2) In the TIME, crosstalk between immune cells and CCSCs forms a “vicious cycle”: where TAMs, Tregs, and others secrete factors like TGF-β/IL-6 to enhance CCSCs stemness, while CCSCs remodel the immunosuppressive microenvironment via exosomes and other pathways (105, 149). (3) Given CCSCs’ plasticity, heterogeneity, and malignant interactions with TIME, single therapeutic strategies prove ineffective. A core strategy to overcome colorectal cancer treatment bottlenecks involves synergistically remodeling TIME through multi-mechanism therapies (150, 151).

Current research on CCSCs has opened new perspectives for colorectal cancer treatment, revealing that these stem cells possess remarkable plasticity and engage in bidirectional malignant interactions with the tumor immune microenvironment. This discovery provides a key breakthrough in elucidating the molecular mechanisms underlying tumor recurrence and metastasis (37). Furthermore, the identification of distinct metastatic tendencies among different CCSCs subtypes offers a basis for developing personalized treatment strategies (10, 152).

Traditional chemotherapy and targeted therapies have significant limitations, while emerging immunotherapies, though promising, remain ineffective against immune “cold tumors.” (153).We conducted an in-depth analysis of the composition of the TIME and its regulatory role in CCSCs. An integrative therapeutic model holds promise to dismantle the survival foundation of CCSCs at their root, offering novel solutions for overcoming treatment resistance and tumor recurrence (154).

However, despite these promising strategic directions, several formidable challenges must be navigated to achieve a breakthrough in controlling CRC recurrence and metastasis. Future research should strategically focus on the following pillars:

First, Decoding Cellular Hierarchies and Identifying CCSC-Specific Markers (155). Leveraging single-cell multi-omics and spatial transcriptomics across treatment timepoints and metastatic sites is essential to determine whether a “master” CCSC subpopulation exists that orchestrates overall heterogeneity, or if this function is distributed among different stem cell clusters. This will identify the most relevant cellular targets and the optimal windows for therapeutic intervention.

Second, Conquering Plasticity through Epigenetic Intervention (156, 157). The regulatory circuitry that drives the “dedifferentiation” of non-stem cells into CCSCs under therapy or immune pressure remains a black box (22). Future work must prioritize elucidating these mechanisms, with a particular emphasis on epigenetic drivers. Identifying key master switches could enable the use of epigenetic modulators to “freeze” CCSCs in a susceptible state or block the regeneration of the stem cell pool, effectively dismantling this critical barrier to treatment (158, 159).

Third, Translating Integrated Therapies into Clinical Practice. The path from laboratory to clinical efficacy for complex combination regimens is fraught with challenges. Future efforts must bridge this gap by developing robust biomarkers to select patients most likely to benefit from specific “offensive-defensive” combinations. A priority is to explore the mechanisms and synergy of an Integrative Therapeutic Model—which combines targeted therapy and chemotherapy to target stemness, immunotherapy to reprogram the TIME (71), and Traditional Chinese Medicine as a multi-targeting adjunct—through robust *in vitro* and *in vivo* studies.

In conclusion, the paradigm for overcoming CRC is decisively shifting from a simplistic “search-and-destroy” mission against CCSCs towards a sophisticated campaign to dismantle the vicious co-evolutionary cycle between the “seed”(CCSCs) and the “soil” (the immunosuppressive TIME). While the challenges are significant, the Integrative Therapeutic Model holds the definitive promise of transforming CRC from a lethal adversary into a manageable chronic condition, and ultimately, achieving the goal of lasting disease control.

## Glossary

5-FU: 5-Fluorouracil
ADCs: Antibody-Drug Conjugates
APC: Adenomatous Polyposis Coli
APM: Antigen Processing Machinery
BiAbs: Bispecific Antibodies
CAFs: Cancer-Associated Fibroblasts
CAR-NK: Chimeric Antigen Receptor Natural Killer cells
CAR-T: Chimeric Antigen Receptor T-cell
CCSCs: Colorectal Cancer Stem Cells
CRC: Colorectal Cancer
CTLA-4: Cytotoxic T-Lymphocyte-Associated Protein 4
DCs: Dendritic Cells
DLL4: Delta-Like Ligand 4
dMMR: Deficient Mismatch Repair
EMT: Epithelial–Mesenchymal Transition
EpCAM: Epithelial Cell Adhesion Molecule
EVs: Extracellular Vesicles
GM-CSF: Granulocyte-Macrophage Colony-Stimulating Factor
G-MDSCs: Granulocyte-derived Myeloid-Derived Suppressor Cells
iNOS: Inducible Nitric Oxide Synthase
ITH: Intratumoral Heterogeneity
MAFA: MAF BZIP Transcription Factor A
MDSCs: Myeloid-Derived Suppressor Cells
MHC: Major Histocompatibility Complex
M-MDSCs: Monocyte-derived Myeloid-Derived Suppressor Cells
MSI-H: Microsatellite Instability-High
MSS: Microsatellite Stable
NETs: Neutrophil Extracellular Traps
NK cells: Natural Killer cells
OnF: Oncogenic Fetal Reprogramming
PAF: Platelet-Activating Factor
PD-1: Programmed Cell Death Protein 1
PD-L1: Programmed Death-Ligand 1
PHGDH: Phosphoglycerate Dehydrogenase
pMMR: Proficient Mismatch Repair
scRNA-seq: Single-cell RNA sequencing
STAT3: Signal Transducer and Activator of Transcription 3
TAA: Tumor-Associated Antigen
TAMs: Tumor-Associated Macrophages
TANs: Tumor-Associated Neutrophils
TCR: T-cell Receptor
TGF-β: Transforming Growth Factor Beta
TIME: Tumor Immune Microenvironment
TMB: Tumor Mutational Burden
TME: Tumor Microenvironment
Tregs: Regulatory T cells
Wnt: Wingless/Integrated.

## References

[1] Xi Y Xu P . Global colorectal cancer burden in 2020 and projections to 2040. Transl Oncol. (2021) 14:101174. doi: 10.1016/j.tranon.2021.101174, PMID: 34243011 PMC8273208

[2] Siegel RL Miller KD Wagle NS Jemal A . Cancer statistics, 2023. CA Cancer J Clin. (2023) 73:17–48. doi: 10.3322/caac.21763, PMID: 36633525

[3] Amin MB Greene FL Edge SB Compton CC Gershenwald JE Brookland RK . The Eighth Edition AJCC Cancer Staging Manual: Continuing to build a bridge from a population-based to a more "personalized" approach to cancer staging. CA Cancer J Clin. (2017) 67:93–9. doi: 10.3322/caac.21388, PMID: 28094848

[4] Wang H Cui G Yu B Sun M Yang H . Cancer stem cell niche in colorectal cancer and targeted therapies. Curr Pharm Des. (2020) 26:1979–93. doi: 10.2174/1381612826666200408102305, PMID: 32268862

[5] Canellas-Socias A Cortina C Hernando-Momblona X Palomo-Ponce S Mulholland EJ Turon G . Metastatic recurrence in colorectal cancer arises from residual EMP1(+) cells. Nature. (2022) 611:603–13. doi: 10.1038/s41586-022-05402-9, PMID: 36352230 PMC7616986

[6] Vitale I Shema E Loi S Galluzzi L . Intratumoral heterogeneity in cancer progression and response to immunotherapy. Nat Med. (2021) 27:212–24. doi: 10.1038/s41591-021-01233-9, PMID: 33574607

[7] Vu T Datta PK . Regulation of EMT in colorectal cancer: A culprit in metastasis. Cancers (Basel). (2017) 9:171. doi: 10.3390/cancers9120171, PMID: 29258163 PMC5742819

[8] Hsu HC Thiam TK Lu YJ Yeh CY Tsai WS You JF . Mutations of KRAS/NRAS/BRAF predict cetuximab resistance in metastatic colorectal cancer patients. Oncotarget. (2016) 7:22257–70. doi: 10.18632/oncotarget.8076, PMID: 26989027 PMC5008360

[9] Blondy S David V Verdier M Mathonnet M Perraud A Christou N . 5-Fluorouracil resistance mechanisms in colorectal cancer: From classical pathways to promising processes. Cancer Sci. (2020) 111:3142–54. doi: 10.1111/cas.14532, PMID: 32536012 PMC7469786

[10] Chao S Zhang F Yan H Wang L Zhang L Wang Z . Targeting intratumor heterogeneity suppresses colorectal cancer chemoresistance and metastasis. EMBO Rep. (2023) 24:e56416. doi: 10.15252/embr.202256416, PMID: 37338390 PMC10398666

[11] Sabini C Sorbi F Cunnea P Fotopoulou C . Ovarian cancer stem cells: ready for prime time? Arch Gynecol Obstet. (2020) 301:895–9. doi: 10.1007/s00404-020-05510-9, PMID: 32200419

[12] Naz F Shi M Sajid S Yang Z Yu C . Cancer stem cells: a major culprit of intra-tumor heterogeneity. Am J Cancer Res. (2021) 11:5782–811. Available online at: https://pubmed.ncbi.nlm.nih.gov/35018226/., PMID: 35018226 PMC8727794

[13] Luo S Yang G Ye P Cao N Chi X Yang WH . Macrophages are a double-edged sword: molecular crosstalk between tumor-associated macrophages and cancer stem cells. Biomolecules. (2022) 12:850. doi: 10.3390/biom12060850, PMID: 35740975 PMC9221070

[14] Zeng X Liu C Yao J Wan H Wan G Li Y . Breast cancer stem cells, heterogeneity, targeting therapies and therapeutic implications. Pharmacol Res. (2021) 163:105320. doi: 10.1016/j.phrs.2020.105320, PMID: 33271295

[15] Grothey A . Pembrolizumab in MSI-H-dMMR advanced colorectal cancer - A new standard of care. N Engl J Med. (2020) 383:2283–5. doi: 10.1056/NEJMe2031294, PMID: 33264550

[16] Du L Cheng Q Zheng H Liu J Liu L Chen Q . Targeting stemness of cancer stem cells to fight colorectal cancers. Semin Cancer Biol. (2022) 82:150–61. doi: 10.1016/j.semcancer.2021.02.012, PMID: 33631296

[17] Barker N Ridgway RA van Es JH van de Wetering M Begthel H van den Born M . Crypt stem cells as the cells-of-origin of intestinal cancer. Nature. (2009) 457:608–11. doi: 10.1038/nature07602, PMID: 19092804

[18] Davis H Irshad S Bansal M Rafferty H Boitsova T Bardella C . Aberrant epithelial GREM1 expression initiates colonic tumorigenesis from cells outside the stem cell niche. Nat Med. (2015) 21:62–70. doi: 10.1038/nm.3750, PMID: 25419707 PMC4594755

[19] Schwitalla S Fingerle AA Cammareri P Nebelsiek T Göktuna SI Ziegler PK . Intestinal tumorigenesis initiated by dedifferentiation and acquisition of stem-cell-like properties. Cell. (2013) 152:25–38. doi: 10.1016/j.cell.2012.12.012, PMID: 23273993

[20] Zeuner A Todaro M Stassi G De Maria R . Colorectal cancer stem cells: from the crypt to the clinic. Cell Stem Cell. (2014) 15:692–705. doi: 10.1016/j.stem.2014.11.012, PMID: 25479747

[21] Chen B Scurrah CR McKinley ET Simmons AJ Ramirez-Solano MA Zhu X . Differential pre-malignant programs and microenvironment chart distinct paths to Malignancy in human colorectal polyps. Cell. (2021) 184:6262–80.e26. doi: 10.1016/j.cell.2021.11.031, PMID: 34910928 PMC8941949

[22] Mzoughi S Schwarz M Wang X Demircioglu D Ulukaya G Mohammed K . Oncofetal reprogramming drives phenotypic plasticity in WNT-dependent colorectal cancer. Nat Genet. (2025) 57:402–12. doi: 10.1038/s41588-024-02058-1, PMID: 39930084 PMC11821538

[23] IJ JEG Dekker E . Gastric metaplasia could initiate the serrated neoplasia pathway in CRC. Nat Rev Gastroenterol Hepatol. (2022) 19:217–8. doi: 10.1038/s41575-022-00592-z, PMID: 35181750

[24] Saw PE Liu Q Wong PP Song E . Cancer stem cell mimicry for immune evasion and therapeutic resistance. Cell Stem Cell. (2024) 31:1101–12. doi: 10.1016/j.stem.2024.06.003, PMID: 38925125

[25] Drost J van Jaarsveld RH Ponsioen B Zimberlin C van Boxtel R Buijs A . Sequential cancer mutations in cultured human intestinal stem cells. Nature. (2015) 521:43–7. doi: 10.1038/nature14415, PMID: 25924068

[26] Lindner P Paul S Eckstein M Hampel C Muenzner JK Erlenbach-Wuensch K . EMT transcription factor ZEB1 alters the epigenetic landscape of colorectal cancer cells. Cell Death Dis. (2020) 11:147. doi: 10.1038/s41419-020-2340-4, PMID: 32094334 PMC7040187

[27] Sipos F Műzes G . Interconnection of CD133 stem cell marker with autophagy and apoptosis in colorectal cancer. Int J Mol Sci. (2024) 25:11201. doi: 10.3390/ijms252011201, PMID: 39456981 PMC11508732

[28] Li R Liu X Huang X Zhang D Chen Z Zhang J . Single-cell transcriptomic analysis deciphers heterogenous cancer stem-like cells in colorectal cancer and their organ-specific metastasis. Gut. (2024) 73:470–84. doi: 10.1136/gutjnl-2023-330243, PMID: 38050068 PMC10894846

[29] Oka T Higa T Sugahara O Koga D Nakayama S Nakayama KI . Ablation of p57+ Quiescent Cancer Stem Cells Suppresses Recurrence after Chemotherapy of Intestinal Tumors. Cancer Res. (2023) 83:1393–409. doi: 10.1158/0008-5472.CAN-22-2578, PMID: 36880956

[30] Shiokawa D Sakai H Ohata H Miyazaki T Kanda Y Sekine S . Slow-cycling cancer stem cells regulate progression and chemoresistance in colon cancer. Cancer Res. (2020) 80:4451–64. doi: 10.1158/0008-5472.CAN-20-0378, PMID: 32816913

[31] Higa T Nakayama KI . Cell cycle heterogeneity and plasticity of colorectal cancer stem cells. Cancer Sci. (2024) 115:1370–7. doi: 10.1111/cas.16117, PMID: 38413370 PMC11093209

[32] Kreso A Dick JE . Evolution of the cancer stem cell model. Cell Stem Cell. (2014) 14:275–91. doi: 10.1016/j.stem.2014.02.006, PMID: 24607403

[33] Bu P Wang L Chen KY Srinivasan T Murthy PK Tung KL . A miR-34a-numb feedforward loop triggered by inflammation regulates asymmetric stem cell division in intestine and colon cancer. Cell Stem Cell. (2016) 18:189–202. doi: 10.1016/j.stem.2016.01.006, PMID: 26849305 PMC4751059

[34] Ayob AZ Ramasamy TS . Cancer stem cells as key drivers of tumour progression. J BioMed Sci. (2018) 25:20. doi: 10.1186/s12929-018-0426-4, PMID: 29506506 PMC5838954

[35] Pérez-González A Bévant K Blanpain C . Cancer cell plasticity during tumor progression, metastasis and response to therapy. Nat Cancer. (2023) 4:1063–82. doi: 10.1038/s43018-023-00595-y, PMID: 37537300 PMC7615147

[36] de Sousa e Melo F Kurtova AV Harnoss JM Kljavin N Hoeck JD Hung J . A distinct role for Lgr5(+) stem cells in primary and metastatic colon cancer. Nature. (2017) 543:676–80. doi: 10.1038/nature21713, PMID: 28358093

[37] Tape CJ . Plastic persisters: revival stem cells in colorectal cancer. Trends Cancer. (2024) 10:185–95. doi: 10.1016/j.trecan.2023.11.003, PMID: 38071119

[38] Vermeulen L Todaro M de Sousa Mello F Sprick MR Kemper K Perez Alea M . Single-cell cloning of colon cancer stem cells reveals a multi-lineage differentiation capacity. Proc Natl Acad Sci U S A. (2008) 105:13427–32. doi: 10.1073/pnas.0805706105, PMID: 18765800 PMC2533206

[39] Moorman A Benitez EK Cambulli F Jiang Q Mahmoud A Lumish M . Progressive plasticity during colorectal cancer metastasis. Nature. (2025) 637:947–54. doi: 10.1038/s41586-024-08150-0, PMID: 39478232 PMC11754107

[40] Shin AE Tesfagiorgis Y Larsen F Derouet M Zeng PYF Good HJ . F4/80(+)Ly6C(high) macrophages lead to cell plasticity and cancer initiation in colitis. Gastroenterology. (2023) 164:593–609.e13. doi: 10.1053/j.gastro.2023.01.002, PMID: 36634827 PMC10038892

[41] Bullock MD Pickard KM Nielsen BS Sayan AE Jenei V Mellone M . Pleiotropic actions of miR-21 highlight the critical role of deregulated stromal microRNAs during colorectal cancer progression. Cell Death Dis. (2013) 4:e684. doi: 10.1038/cddis.2013.213, PMID: 23788041 PMC3702298

[42] Reinsalu L Puurand M Chekulayev V Miller S Shevchuk I Tepp K . Energy metabolic plasticity of colorectal cancer cells as a determinant of tumor growth and metastasis. Front Oncol. (2021) 11:698951. doi: 10.3389/fonc.2021.698951, PMID: 34381722 PMC8351413

[43] Hao Y Samuels Y Li Q Krokowski D Guan BJ Wang C . Oncogenic PIK3CA mutations reprogram glutamine metabolism in colorectal cancer. Nat Commun. (2016) 7:11971. doi: 10.1038/ncomms11971, PMID: 27321283 PMC4915131

[44] Erazo-Oliveras A Muñoz-Vega M Mlih M Thiriveedi V Salinas ML Rivera-Rodríguez JM . Mutant APC reshapes Wnt signaling plasma membrane nanodomains by altering cholesterol levels *via* oncogenic β-catenin. Nat Commun. (2023) 14:4342. doi: 10.1038/s41467-023-39640-w, PMID: 37468468 PMC10356786

[45] Lin K Chowdhury S Zeineddine MA Zeineddine FA Hornstein NJ Villarreal OE . Identification of colorectal cancer cell stemness from single-cell RNA sequencing. Mol Cancer Res. (2024) 22:337–46. doi: 10.1158/1541-7786.MCR-23-0468, PMID: 38156967 PMC10987274

[46] Kemper K Prasetyanti PR De Lau W Rodermond H Clevers H Medema JP . Monoclonal antibodies against Lgr5 identify human colorectal cancer stem cells. Stem Cells. (2012) 30:2378–86. doi: 10.1002/stem.1233, PMID: 22969042

[47] Wang W Lokman NA Barry SC Oehler MK Ricciardelli C . LGR5: An emerging therapeutic target for cancer metastasis and chemotherapy resistance. Cancer Metastasis Rev. (2025) 44:23. doi: 10.1007/s10555-024-10239-x, PMID: 39821694 PMC11742290

[48] Huang JL Oshi M Endo I Takabe K . Clinical relevance of stem cell surface markers CD133, CD24, and CD44 in colorectal cancer. Am J Cancer Res. (2021) 11:5141–54. Available online at: https://pubmed.ncbi.nlm.nih.gov/34765317/., PMID: 34765317 PMC8569346

[49] Kostovski O Jovanovikj R Kostovska I . Co-expression of stem cell markers CD133 and CD44 as predictors of metastatic potential of colorectal carcinoma. Turk J Surg. (2025) 41:174–9. doi: 10.47717/turkjsurg.2025.6837, PMID: 40452184 PMC12124339

[50] Yang Y Zhu G Yang L Yang Y . Targeting CD24 as a novel immunotherapy for solid cancers. Cell Commun Signal. (2023) 21:312. doi: 10.1186/s12964-023-01315-w, PMID: 37919766 PMC10623753

[51] Zhao Q Zong H Zhu P Su C Tang W Chen Z . Crosstalk between colorectal CSCs and immune cells in tumorigenesis, and strategies for targeting colorectal CSCs. Exp Hematol Oncol. (2024) 13:6. doi: 10.1186/s40164-024-00474-x, PMID: 38254219 PMC10802076

[52] Leng Z Xia Q Chen J Li Y Xu J Zhao E . Lgr5+CD44+EpCAM+ Strictly defines cancer stem cells in human colorectal cancer. Cell Physiol Biochem. (2018) 46:860–72. doi: 10.1159/000488743, PMID: 29627827

[53] Gires O Pan M Schinke H Canis M Baeuerle PA . Expression and function of epithelial cell adhesion molecule EpCAM: where are we after 40 years? Cancer Metastasis Rev. (2020) 39:969–87. doi: 10.1007/s10555-020-09898-3, PMID: 32507912 PMC7497325

[54] GaGhana LO Miskad U Cangara MH Zainuddin AA Mardiati M Arsyadi G . The relationship between expression of epCAM cancer stem cell marker with histopathological grading, lymphovascular invasion, and metastases in colorectal adenocarcinoma. Asian Pac J Cancer Prev. (2023) 24:929–34. doi: 10.31557/APJCP.2023.24.3.929, PMID: 36974547 PMC10334088

[55] Takeda T Yokoyama Y Takahashi H Okuzaki D Asai K Itakura H . A stem cell marker KLF5 regulates CCAT1 *via* three-dimensional genome structure in colorectal cancer cells. Br J Cancer. (2022) 126:109–19. doi: 10.1038/s41416-021-01579-4, PMID: 34707247 PMC8727571

[56] Babajnai A Rahmani S Asadi MJ Gheytanchi E Adibhesami G Vakhshiteh F . Molecular and phenotypic characterization of 5-FU resistant colorectal cancer cells: toward enrichment of cancer stem cells. Cancer Cell Int. (2025) 25:154. doi: 10.1186/s12935-025-03758-2, PMID: 40251609 PMC12008981

[57] Saigusa S Tanaka K Toiyama Y Yokoe T Okugawa Y Ioue Y . Correlation of CD133, OCT4, and SOX2 in rectal cancer and their association with distant recurrence after chemoradiotherapy. Ann Surg Oncol. (2009) 16:3488–98. doi: 10.1245/s10434-009-0617-z, PMID: 19657699

[58] Meng HM Zheng P Wang XY Liu C Sui HM Wu SJ . Over-expression of Nanog predicts tumor progression and poor prognosis in colorectal cancer. Cancer Biol Ther. (2010) 9:295–302. doi: 10.4161/cbt.9.4.10666, PMID: 20026903

[59] Tan L Peng D Cheng Y . Significant position of C-myc in colorectal cancer: a promising therapeutic target. Clin Transl Oncol. (2022) 24:2295–304. doi: 10.1007/s12094-022-02910-y, PMID: 35972682

[60] Kreso A van Galen P Pedley NM Lima-Fernandes E Frelin C Davis T . Self-renewal as a therapeutic target in human colorectal cancer. Nat Med. (2014) 20:29–36. doi: 10.1038/nm.3418, PMID: 24292392

[61] Hsu YC Luo CW Huang WL Wu CC Chou CL Chen CI . BMI1-KLF4 axis deficiency improves responses to neoadjuvant concurrent chemoradiotherapy in patients with rectal cancer. Radiother Oncol. (2020) 149:249–58. doi: 10.1016/j.radonc.2020.06.023, PMID: 32592893

[62] Merlos-Suárez A Barriga FM Jung P Iglesias M Céspedes MV Rossell D . The intestinal stem cell signature identifies colorectal cancer stem cells and predicts disease relapse. Cell Stem Cell. (2011) 8:511–24. doi: 10.1016/j.stem.2011.02.020, PMID: 21419747

[63] Sipos F Műzes G . Cancer stem cell relationship with pro-tumoral inflammatory microenvironment. Biomedicines. (2023) 11:189. doi: 10.3390/biomedicines11010189, PMID: 36672697 PMC9855358

[64] Wang QH Zhang M Shi CT Xie JJ Chen F Shi QF . High Oct4 predicted worse prognosis of right-sided colon cancer patients. Future Oncol. (2018) 14:2279–91. doi: 10.2217/fon-2018-0046, PMID: 29656661

[65] Fujino S Miyoshi N . Oct4 gene expression in primary colorectal cancer promotes liver metastasis. Stem Cells Int. (2019) 2019:7896524. doi: 10.1155/2019/7896524, PMID: 31191684 PMC6525814

[66] Hua F Shang S Yang YW Zhang HZ Xu TL Yu JJ . TRIB3 interacts with β-catenin and TCF4 to increase stem cell features of colorectal cancer stem cells and tumorigenesis. Gastroenterology. (2019) 156:708–21.e15. doi: 10.1053/j.gastro.2018.10.031, PMID: 30365932

[67] Zhou X Xie X Liu T Chen S Wang Y Zhang J . REC8 enhances stemness and promotes metastasis of colorectal cancer through BTK/Akt/β-catenin signaling pathway. Transl Oncol. (2022) 15:101305. doi: 10.1016/j.tranon.2021.101305, PMID: 34890967 PMC8662335

[68] Wang M Tang L Chen S Wang L Wu J Zhong C . ZNF217-activated Notch signaling mediates sulforaphane-suppressed stem cell properties in colorectal cancer. J Nutr Biochem. (2024) 125:109551. doi: 10.1016/j.jnutbio.2023.109551, PMID: 38134973

[69] Du L Rao G Wang H Li B Tian W Cui J . CD44-positive cancer stem cells expressing cellular prion protein contribute to metastatic capacity in colorectal cancer. Cancer Res. (2013) 73:2682–94. doi: 10.1158/0008-5472.CAN-12-3759, PMID: 23418321

[70] Morral C Stanisavljevic J Hernando-Momblona X Mereu E Álvarez-Varela A Cortina C . Zonation of ribosomal DNA transcription defines a stem cell hierarchy in colorectal cancer. Cell Stem Cell. (2020) 26:845–61.e12. doi: 10.1016/j.stem.2020.04.012, PMID: 32396863 PMC9006079

[71] Tauriello DVF Palomo-Ponce S Stork D Berenguer-Llergo A Badia-Ramentol J Iglesias M . TGFβ drives immune evasion in genetically reconstituted colon cancer metastasis. Nature. (2018) 554:538–43. doi: 10.1038/nature25492, PMID: 29443964

[72] Zhang L Yu X Zheng L Zhang Y Li Y Fang Q . Lineage tracking reveals dynamic relationships of T cells in colorectal cancer. Nature. (2018) 564:268–72. doi: 10.1038/s41586-018-0694-x, PMID: 30479382

[73] Gajewski TF Schreiber H Fu YX . Innate and adaptive immune cells in the tumor microenvironment. Nat Immunol. (2013) 14:1014–22. doi: 10.1038/ni.2703, PMID: 24048123 PMC4118725

[74] Yu J Yan Y Li S Xu Y Parolia A Rizvi S . Progestogen-driven B7-H4 contributes to onco-fetal immune tolerance. Cell. (2024) 187:4713–32.e19. doi: 10.1016/j.cell.2024.06.012, PMID: 38968937 PMC11344674

[75] Volonté A Di Tomaso T Spinelli M Todaro M Sanvito F Albarello L . Cancer-initiating cells from colorectal cancer patients escape from T cell-mediated immunosurveillance *in vitro* through membrane-bound IL-4. J Immunol. (2014) 192:523–32. doi: 10.4049/jimmunol.1301342, PMID: 24277698 PMC8744948

[76] Chou C Zhang X Krishna C Nixon BG Dadi S Capistrano KJ . Programme of self-reactive innate-like T cell-mediated cancer immunity. Nature. (2022) 605:139–45. doi: 10.1038/s41586-022-04632-1, PMID: 35444279 PMC9250102

[77] Goto N Westcott PMK Goto S Imada S Taylor MS Eng G . SOX17 enables immune evasion of early colorectal adenomas and cancers. Nature. (2024) 627:636–45. doi: 10.1038/s41586-024-07135-3, PMID: 38418875 PMC11969226

[78] Agudo J Miao Y . Stemness in solid Malignancies: coping with immune attack. Nat Rev Cancer. (2025) 25:27–40. doi: 10.1038/s41568-024-00760-0, PMID: 39455862

[79] Jewett A Tseng HC Arasteh A Saadat S Christensen RE Cacalano NA . Natural killer cells preferentially target cancer stem cells; role of monocytes in protection against NK cell mediated lysis of cancer stem cells. Curr Drug Deliv. (2012) 9:5–16. doi: 10.2174/156720112798375989, PMID: 22023212

[80] Koh J Lee SB Park H Lee HJ Cho NH Kim J . Susceptibility of CD24(+) ovarian cancer cells to anti-cancer drugs and natural killer cells. Biochem Biophys Res Commun. (2012) 427:373–8. doi: 10.1016/j.bbrc.2012.09.067, PMID: 22995296

[81] Ames E Canter RJ Grossenbacher SK Mac S Chen M Smith RC . NK cells preferentially target tumor cells with a cancer stem cell phenotype. J Immunol. (2015) 195:4010–9. doi: 10.4049/jimmunol.1500447, PMID: 26363055 PMC4781667

[82] Kang JH Zappasodi R . Modulating Treg stability to improve cancer immunotherapy. Trends Cancer. (2023) 9:911–27. doi: 10.1016/j.trecan.2023.07.015, PMID: 37598003 PMC13390109

[83] Taylor A Verhagen J Blaser K Akdis M Akdis CA . Mechanisms of immune suppression by interleukin-10 and transforming growth factor-beta: the role of T regulatory cells. Immunology. (2006) 117:433–42. doi: 10.1111/j.1365-2567.2006.02321.x, PMID: 16556256 PMC1782242

[84] Shiri AM Zhang T Bedke T Zazara DE Zhao L Lucke J . IL-10 dampens antitumor immunity and promotes liver metastasis *via* PD-L1 induction. J Hepatol. (2024) 80:634–44. doi: 10.1016/j.jhep.2023.12.015, PMID: 38160941 PMC10964083

[85] Oderup C Cederbom L Makowska A Cilio CM Ivars F . Cytotoxic T lymphocyte antigen-4-dependent down-modulation of costimulatory molecules on dendritic cells in CD4+ CD25+ regulatory T-cell-mediated suppression. Immunology. (2006) 118:240–9. doi: 10.1111/j.1365-2567.2006.02362.x, PMID: 16771859 PMC1782280

[86] Kono K Kawaida H Takahashi A Sugai H Mimura K Miyagawa N . CD4(+)CD25high regulatory T cells increase with tumor stage in patients with gastric and esophageal cancers. Cancer Immunol Immunother. (2006) 55:1064–71. doi: 10.1007/s00262-005-0092-8, PMID: 16328385 PMC11030626

[87] Wculek SK Cueto FJ Mujal AM Melero I Krummel MF Sancho D . Dendritic cells in cancer immunology and immunotherapy. Nat Rev Immunol. (2020) 20:7–24. doi: 10.1038/s41577-019-0210-z, PMID: 31467405

[88] Tai Y Chen M Wang F Fan Y Zhang J Cai B . The role of dendritic cells in cancer immunity and therapeutic strategies. Int Immunopharmacol. (2024) 128:111548. doi: 10.1016/j.intimp.2024.111548, PMID: 38244518

[89] Szaryńska M Olejniczak A Kobiela J Łaski D Śledziński Z Kmieć Z . Cancer stem cells as targets for DC-based immunotherapy of colorectal cancer. Sci Rep. (2018) 8:12042. doi: 10.1038/s41598-018-30525-3, PMID: 30104575 PMC6089981

[90] Wang J Zhao X Wan YY . Intricacies of TGF-β signaling in Treg and Th17 cell biology. Cell Mol Immunol. (2023) 20:1002–22. doi: 10.1038/s41423-023-01036-7, PMID: 37217798 PMC10468540

[91] Carlini V Noonan DM Abdalalem E Goletti D Sansone C Calabrone L . The multifaceted nature of IL-10: regulation, role in immunological homeostasis and its relevance to cancer, COVID-19 and post-COVID conditions. Front Immunol. (2023) 14. doi: 10.3389/fimmu.2023.1161067, PMID: 37359549 PMC10287165

[92] Fan C Wu J Shen Y Hu H Wang Q Mao Y . Hypoxia promotes the tolerogenic phenotype of plasmacytoid dendritic cells in head and neck squamous cell carcinoma. Cancer Med. (2022) 11:922–30. doi: 10.1002/cam4.4511, PMID: 34964283 PMC8855917

[93] Węgierek-Ciura K Mierzejewska J Szczygieł A Rossowska J Wróblewska A Świtalska M . Inhibition of MC38 colon cancer growth by multicomponent chemoimmunotherapy with anti-IL-10R antibodies, HES-MTX nanoconjugate, depends on application of IL-12, IL-15 or IL-18 secreting dendritic cell vaccines. Front Immunol. (2023) 14:1212606. doi: 10.3389/fimmu.2023.1212606, PMID: 37545526 PMC10399586

[94] Binnewies M Mujal AM Pollack JL Combes AJ Hardison EA Barry KC . Unleashing type-2 dendritic cells to drive protective antitumor CD4(+) T cell immunity. Cell. (2019) 177:556–71.e16. doi: 10.1016/j.cell.2019.02.005, PMID: 30955881 PMC6954108

[95] Moreno Ayala MA Campbell TF Zhang C Dahan N Bockman A Prakash V . CXCR3 expression in regulatory T cells drives interactions with type I dendritic cells in tumors to restrict CD8(+) T cell antitumor immunity. Immunity. (2023) 56:1613–30.e5. doi: 10.1016/j.immuni.2023.06.003, PMID: 37392735 PMC10752240

[96] You S Li S Zeng L Song J Li Z Li W . Lymphatic-localized Treg-mregDC crosstalk limits antigen trafficking and restrains anti-tumor immunity. Cancer Cell. (2024) 42:1415–33.e12. doi: 10.1016/j.ccell.2024.06.014, PMID: 39029466

[97] Maldonado RA von Andrian UH . How tolerogenic dendritic cells induce regulatory T cells. Adv Immunol. (2010) 108:111–65. doi: 10.1016/B978-0-12-380995-7.00004-5, PMID: 21056730 PMC3050492

[98] Bain CC Mowat AM . Macrophages in intestinal homeostasis and inflammation. Immunol Rev. (2014) 260:102–17. doi: 10.1111/imr.12192, PMID: 24942685 PMC4141699

[99] Kawano Y Nakae J Watanabe N Kikuchi T Tateya S Tamori Y . Colonic pro-inflammatory macrophages cause insulin resistance in an intestinal ccl2/ccr2-dependent manner. Cell Metab. (2016) 24:295–310. doi: 10.1016/j.cmet.2016.07.009, PMID: 27508875

[100] Wang H Tian T Zhang J . Tumor-associated macrophages (TAMs) in colorectal cancer (CRC): from mechanism to therapy and prognosis. Int J Mol Sci. (2021) 22:8470. doi: 10.3390/ijms22168470, PMID: 34445193 PMC8395168

[101] Zhang Q Sioud M . Tumor-associated macrophage subsets: shaping polarization and targeting. Int J Mol Sci. (2023) 24:7493. doi: 10.3390/ijms24087493, PMID: 37108657 PMC10138703

[102] Ngambenjawong C Gustafson HH Pun SH . Progress in tumor-associated macrophage (TAM)-targeted therapeutics. Adv Drug Delivery Rev. (2017) 114:206–21. doi: 10.1016/j.addr.2017.04.010, PMID: 28449873 PMC5581987

[103] Malfitano AM Pisanti S Napolitano F Di Somma S Martinelli R Portella G . Tumor-associated macrophage status in cancer treatment. Cancers (Basel). (2020) 12:1987. doi: 10.3390/cancers12071987, PMID: 32708142 PMC7409350

[104] Pan Y Yu Y Wang X Zhang T . Tumor-associated macrophages in tumor immunity. Front Immunol. (2020) 11:583084. doi: 10.3389/fimmu.2020.583084, PMID: 33365025 PMC7751482

[105] Huang YJ Yang CK Wei PL Huynh TT Whang-Peng J Meng TC . Ovatodiolide suppresses colon tumorigenesis and prevents polarization of M2 tumor-associated macrophages through YAP oncogenic pathways. J Hematol Oncol. (2017) 10:60. doi: 10.1186/s13045-017-0421-3, PMID: 28241877 PMC5329923

[106] Lei MML Lee TKW . Cancer stem cells: emerging key players in immune evasion of cancers. Front Cell Dev Biol. (2021) 9:692940. doi: 10.3389/fcell.2021.692940, PMID: 34235155 PMC8257022

[107] Shang S Yang C Chen F Xiang RS Zhang H Dai SY . ID1 expressing macrophages support cancer cell stemness and limit CD8(+) T cell infiltration in colorectal cancer. Nat Commun. (2023) 14:7661. doi: 10.1038/s41467-023-43548-w, PMID: 37996458 PMC10667515

[108] Francipane MG Alea MP Lombardo Y Todaro M Medema JP Stassi G . Crucial role of interleukin-4 in the survival of colon cancer stem cells. Cancer Res. (2008) 68:4022–5. doi: 10.1158/0008-5472.CAN-07-6874, PMID: 18519657

[109] Cai Z Li W Hager S Wilson JL Afjehi-Sadat L Heiss EH . Targeting PHGDH reverses the immunosuppressive phenotype of tumor-associated macrophages through alpha-ketoglutarate and mTORC1 signaling. Cell Mol Immunol. (2024) 21:448–65. doi: 10.1038/s41423-024-01134-0, PMID: 38409249 PMC11061172

[110] Kerneur C Cano CE Olive D . Major pathways involved in macrophage polarization in cancer. Front Immunol. (2022) 13:1026954. doi: 10.3389/fimmu.2022.1026954, PMID: 36325334 PMC9618889

[111] Wang D Wang X Si M Yang J Sun S Wu H . Exosome-encapsulated miRNAs contribute to CXCL12/CXCR4-induced liver metastasis of colorectal cancer by enhancing M2 polarization of macrophages. Cancer Lett. (2020) 474:36–52. doi: 10.1016/j.canlet.2020.01.005, PMID: 31931030

[112] Colangelo T Polcaro G Muccillo L D'Agostino G Rosato V Ziccardi P . Friend or foe? The tumour microenvironment dilemma in colorectal cancer. Biochim Biophys Acta Rev Cancer. (2017) 1867:1–18. doi: 10.1016/j.bbcan.2016.11.001, PMID: 27864070

[113] Gungabeesoon J Gort-Freitas NA Kiss M Bolli E Messemaker M Siwicki M . A neutrophil response linked to tumor control in immunotherapy. Cell. (2023) 186:1448–64.e20. doi: 10.1016/j.cell.2023.02.032, PMID: 37001504 PMC10132778

[114] Peddareddigari VG Wang D Dubois RN . The tumor microenvironment in colorectal carcinogenesis. Cancer Microenviron. (2010) 3:149–66. doi: 10.1007/s12307-010-0038-3, PMID: 21209781 PMC2990487

[115] Stehr AM Wang G Demmler R Stemmler MP Krug J Tripal P . Neutrophil extracellular traps drive epithelial-mesenchymal transition of human colon cancer. J Pathol. (2022) 256:455–67. doi: 10.1002/path.5860, PMID: 34939675

[116] Hwang WL Lan HY Cheng WC Huang SC Yang MH . Tumor stem-like cell-derived exosomal RNAs prime neutrophils for facilitating tumorigenesis of colon cancer. J Hematol Oncol. (2019) 12:10. doi: 10.1186/s13045-019-0699-4, PMID: 30683126 PMC6347849

[117] Linde IL Prestwood TR Qiu J Pilarowski G Linde MH Zhang X . Neutrophil-activating therapy for the treatment of cancer. Cancer Cell. (2023) 41:356–72.e10. doi: 10.1016/j.ccell.2023.01.002, PMID: 36706760 PMC9968410

[118] Jaillon S Ponzetta A Di Mitri D Santoni A Bonecchi R Mantovani A . Neutrophil diversity and plasticity in tumour progression and therapy. Nat Rev Cancer. (2020) 20:485–503. doi: 10.1038/s41568-020-0281-y, PMID: 32694624

[119] Qin F Liu X Chen J Huang S Wei W Zou Y . Anti-TGF-β attenuates tumor growth *via* polarization of tumor associated neutrophils towards an anti-tumor phenotype in colorectal cancer. J Cancer. (2020) 11:2580–92. doi: 10.7150/jca.38179, PMID: 32201528 PMC7066015

[120] Sounbuli K Mironova N Alekseeva L . Diverse neutrophil functions in cancer and promising neutrophil-based cancer therapies. Int J Mol Sci. (2022) 23:15827. doi: 10.3390/ijms232415827, PMID: 36555469 PMC9779721

[121] Deng H Lin C Garcia-Gerique L Fu S Cruz Z Bonner EE . A novel selective inhibitor JBI-589 targets PAD4-mediated neutrophil migration to suppress tumor progression. Cancer Res. (2022) 82:3561–72. doi: 10.1158/0008-5472.CAN-21-4045, PMID: 36069973 PMC9532374

[122] Adrover JM McDowell SAC He XY Quail DF Egeblad M . NETworking with cancer: The bidirectional interplay between cancer and neutrophil extracellular traps. Cancer Cell. (2023) 41:505–26. doi: 10.1016/j.ccell.2023.02.001, PMID: 36827980 PMC10280682

[123] Liang H Du Y Zhu C Zhang Z Liao G Liu L . Nanoparticulate cationic poly(amino acid)s block cancer metastases by destructing neutrophil extracellular traps. ACS Nano. (2023) 17:2868–80. doi: 10.1021/acsnano.2c11280, PMID: 36648411

[124] Hegde S Leader AM Merad M . MDSC: Markers, development, states, and unaddressed complexity. Immunity. (2021) 54:875–84. doi: 10.1016/j.immuni.2021.04.004, PMID: 33979585 PMC8709560

[125] Veglia F Sanseviero E Gabrilovich DI . Myeloid-derived suppressor cells in the era of increasing myeloid cell diversity. Nat Rev Immunol. (2021) 21:485–98. doi: 10.1038/s41577-020-00490-y, PMID: 33526920 PMC7849958

[126] Piqueras-Nebot M Benet M Estors M Cremades A Juan-Vidal Ó Carretero J . A novel method for isolation of tumor infiltrating myeloid-derived suppressor cells from human lung tumor tissue. Sci Rep. (2025) 15:15175. doi: 10.1038/s41598-025-99877-x, PMID: 40307421 PMC12044027

[127] Law AMK Valdes-Mora F Gallego-Ortega D . Myeloid-derived suppressor cells as a therapeutic target for cancer. Cells. (2020) 9:561. doi: 10.3390/cells9030561, PMID: 32121014 PMC7140518

[128] Wang Y Yin K Tian J Xia X Ma J Tang X . Granulocytic myeloid-derived suppressor cells promote the stemness of colorectal cancer cells through exosomal S100A9. Adv Sci (Weinh). (2019) 6:1901278. doi: 10.1002/advs.201901278, PMID: 31559140 PMC6755519

[129] Lau EY Ho NP Lee TK . Cancer stem cells and their microenvironment: biology and therapeutic implications. Stem Cells Int. (2017) 2017:3714190. doi: 10.1155/2017/3714190, PMID: 28337221 PMC5346399

[130] Inamoto S Itatani Y Yamamoto T Minamiguchi S Hirai H Iwamoto M . Loss of SMAD4 promotes colorectal cancer progression by accumulation of myeloid-derived suppressor cells through the CCL15-CCR1 chemokine axis. Clin Cancer Res. (2016) 22:492–501. doi: 10.1158/1078-0432.CCR-15-0726, PMID: 26341919

[131] Wang Y Liu H Zhang Z Bian D Shao K Wang S . G-MDSC-derived exosomes mediate the differentiation of M-MDSC into M2 macrophages promoting colitis-to-cancer transition. J Immunother Cancer. (2023) 11:e006166. doi: 10.1136/jitc-2022-006166, PMID: 37364932 PMC10410840

[132] Cho YH Ro EJ Yoon JS Mizutani T Kang DW Park JC . 5-FU promotes stemness of colorectal cancer *via* p53-mediated WNT/β-catenin pathway activation. Nat Commun. (2020) 11:5321. doi: 10.1038/s41467-020-19173-2, PMID: 33087710 PMC7578039

[133] Tsoumas D Nikou S Giannopoulou E Champeris Tsaniras S Sirinian C Maroulis I . ILK expression in colorectal cancer is associated with EMT, cancer stem cell markers and chemoresistance. Cancer Genomics Proteomics. (2018) 15:127–41. doi: 10.21873/cgp.20071, PMID: 29496692 PMC5892607

[134] Chen Y Yang Z He X Zhu W Wang Y Li J . Proanthocyanidins inhibited colorectal cancer stem cell characteristics through Wnt/β-catenin signaling. Environ Toxicol. (2023) 38:2894–903. doi: 10.1002/tox.23924, PMID: 37551626

[135] Madan B Ke Z Harmston N Ho SY Frois AO Alam J . Wnt addiction of genetically defined cancers reversed by PORCN inhibition. Oncogene. (2016) 35:2197–207. doi: 10.1038/onc.2015.280, PMID: 26257057 PMC4650263

[136] Storm EE Durinck S de Sousa e Melo F Tremayne J Kljavin N Tan C . Targeting PTPRK-RSPO3 colon tumours promotes differentiation and loss of stem-cell function. Nature. (2016) 529:97–100. doi: 10.1038/nature16466, PMID: 26700806

[137] Mariathasan S Turley SJ Nickles D Castiglioni A Yuen K Wang Y . TGFβ attenuates tumour response to PD-L1 blockade by contributing to exclusion of T cells. Nature. (2018) 554:544–8. doi: 10.1038/nature25501, PMID: 29443960 PMC6028240

[138] Zheng F Zhang S Chang AE Moon JJ Wicha MS Wang SX . Breaking immunosuppression to enhance cancer stem cell-targeted immunotherapy. Int J Biol Sci. (2025) 21:1819–36. doi: 10.7150/ijbs.101025, PMID: 39990669 PMC11844285

[139] Li L Jensen RA . Understanding and overcoming immunosuppression shaped by cancer stem cells. Cancer Res. (2023) 83:2096–104. doi: 10.1158/0008-5472.CAN-23-0230, PMID: 37403628 PMC10320482

[140] Clifton GT Rothenberg M Ascierto PA Begley G Cecchini M Eder JP . Developing a definition of immune exclusion in cancer: results of a modified Delphi workshop. J Immunother Cancer. (2023) 11:e006773. doi: 10.1136/jitc-2023-006773, PMID: 37290925 PMC10254706

[141] Tomasian A Jennings JW . Hot and cold spine tumor ablations. Neuroimaging Clin N Am. (2019) 29:529–38. doi: 10.1016/j.nic.2019.07.001, PMID: 31677728

[142] Mulet-Margalef N Linares J Badia-Ramentol J Jimeno M Sanz Monte C Manzano Mozo JL . Challenges and therapeutic opportunities in the dMMR/MSI-H colorectal cancer landscape. Cancers (Basel). (2023) 15:1022. doi: 10.3390/cancers15041022, PMID: 36831367 PMC9954007

[143] Kim GR Ha GH Bae JH Oh SO Kim SH Kang CD . Metastatic colon cancer cell populations contain more cancer stem-like cells with a higher susceptibility to natural killer cell-mediated lysis compared with primary colon cancer cells. Oncol Lett. (2015) 9:1641–6. doi: 10.3892/ol.2015.2918, PMID: 25789015 PMC4356422

[144] Chen HC Mueller N Stott K Kapeni C Rivers E Sauer CM . Novel immunotherapeutics against LGR5 to target multiple cancer types. EMBO Mol Med. (2024) 16:2233–61. doi: 10.1038/s44321-024-00121-2, PMID: 39169164 PMC11393416

[145] Bagus BI . Autologous natural killer cells as a promising immunotherapy for locally advanced colon adenocarcinoma: Three years follow-up of resectable case. Cancer Rep (Hoboken). (2023) 6:e1866. doi: 10.1002/cnr2.1866, PMID: 37439389 PMC10480413

[146] Sun Q Hong Z Zhang C Wang L Han Z Ma D . Immune checkpoint therapy for solid tumours: clinical dilemmas and future trends. Signal Transduct Target Ther. (2023) 8:320. doi: 10.1038/s41392-023-01522-4, PMID: 37635168 PMC10460796

[147] Liao W Zhang L Chen X Xiang J Zheng Q Chen N . Targeting cancer stem cells and signalling pathways through phytochemicals: A promising approach against colorectal cancer. Phytomedicine. (2023) 108:154524. doi: 10.1016/j.phymed.2022.154524, PMID: 36375238

[148] Lepore Signorile M Di Nicola E Forte G Sanese P Fasano C Disciglio V . Tailoring a novel colorectal cancer stem cell-targeted therapy by inhibiting the SMYD3/c-MYC axis. Signal Transduct Target Ther. (2025) 10:206. doi: 10.1038/s41392-025-02290-z, PMID: 40588481 PMC12209437

[149] Novoa Díaz MB Carriere P Gentili C . How the interplay among the tumor microenvironment and the gut microbiota influences the stemness of colorectal cancer cells. World J Stem Cells. (2023) 15:281–301. doi: 10.4252/wjsc.v15.i5.281, PMID: 37342226 PMC10277969

[150] Guo G Tan Z Liu Y Shi F She J . The therapeutic potential of stem cell-derived exosomes in the ulcerative colitis and colorectal cancer. Stem Cell Res Ther. (2022) 13:138. doi: 10.1186/s13287-022-02811-5, PMID: 35365226 PMC8973885

[151] Abedizadeh R Majidi F Khorasani HR Abedi H Sabour D . Colorectal cancer: a comprehensive review of carcinogenesis, diagnosis, and novel strategies for classified treatments. Cancer Metastasis Rev. (2024) 43:729–53. doi: 10.1007/s10555-023-10158-3, PMID: 38112903

[152] Zhang H Zhuo C Lin R Ke F Wang M Yang C . Identification and verification of key genes in colorectal cancer liver metastases through analysis of single-cell sequencing data and TCGA data. Ann Surg Oncol. (2024) 31:8664–79. doi: 10.1245/s10434-024-16194-9, PMID: 39382748 PMC11549235

[153] Zheng H Liu H Li H Dou W Wang J Zhang J . Characterization of stem cell landscape and identification of stemness-relevant prognostic gene signature to aid immunotherapy in colorectal cancer. Stem Cell Res Ther. (2022) 13:244. doi: 10.1186/s13287-022-02913-0, PMID: 35681225 PMC9185878

[154] Nie X Liu H Ye W Wei X Fan L Ma H . LRP5 promotes cancer stem cell traits and chemoresistance in colorectal cancer. J Cell Mol Med. (2022) 26:1095–112. doi: 10.1111/jcmm.17164, PMID: 34997691 PMC8831954

[155] Soleimani A Saeedi N Al-Asady AM Nazari E Hanaie R Khazaei M . Colorectal cancer stem cell biomarkers: biological traits and prognostic insights. Curr Pharm Des. (2024) 30:1386–97. doi: 10.2174/0113816128291321240329050945, PMID: 38623972

[156] Liang X Duronio GN Yang Y Bala P Hebbar P Spisak S . An enhancer-driven stem cell-like program mediated by SOX9 blocks intestinal differentiation in colorectal cancer. Gastroenterology. (2022) 162:209–22. doi: 10.1053/j.gastro.2021.09.044, PMID: 34571027 PMC10035046

[157] Wang Q Hu T Zhang Q Zhang Y Dong X Jin Y . Fusobacterium nucleatum promotes colorectal cancer through neogenesis of tumor stem cells. J Clin Invest. (2025) 135:e181595. doi: 10.1172/JCI181595, PMID: 39656543 PMC11785920

[158] Underwood PW Ruff SM Pawlik TM . Update on targeted therapy and immunotherapy for metastatic colorectal cancer. Cells. (2024) 13:245. doi: 10.3390/cells13030245, PMID: 38334637 PMC10854977

[159] Singh M Morris VK Bandey IN Hong DS Kopetz S . Advancements in combining targeted therapy and immunotherapy for colorectal cancer. Trends Cancer. (2024) 10:598–609. doi: 10.1016/j.trecan.2024.05.001, PMID: 38821852

